# Patterns of Transcriptional Response to 1,25-Dihydroxyvitamin D3 and Bacterial Lipopolysaccharide in Primary Human Monocytes

**DOI:** 10.1534/g3.116.028712

**Published:** 2016-03-11

**Authors:** Silvia N. Kariuki, John D. Blischak, Shigeki Nakagome, David B. Witonsky, Anna Di Rienzo

**Affiliations:** Department of Human Genetics, University of Chicago, Illinois 60637

**Keywords:** differential expression profiling, innate immune cells, proinflammatory response, pathway analysis

## Abstract

The active form of vitamin D, 1,25-dihydroxyvitamin D3 (1,25D), plays an important immunomodulatory role, regulating transcription of genes in the innate and adaptive immune system. The present study examines patterns of transcriptome-wide response to 1,25D, and the bacterial lipopolysaccharide (LPS) in primary human monocytes, to elucidate pathways underlying the effects of 1,25D on the immune system. Monocytes obtained from healthy individuals of African-American and European-American ancestry were treated with 1,25D, LPS, or both, simultaneously. The addition of 1,25D during stimulation with LPS induced significant upregulation of genes in the antimicrobial and autophagy pathways, and downregulation of proinflammatory response genes compared to LPS treatment alone. A joint Bayesian analysis enabled clustering of genes into patterns of shared transcriptional response across treatments. The biological pathways enriched within these expression patterns highlighted several mechanisms through which 1,25D could exert its immunomodulatory role. Pathways such as mTOR signaling, EIF2 signaling, IL-8 signaling, and Tec Kinase signaling were enriched among genes with opposite transcriptional responses to 1,25D and LPS, respectively, highlighting the important roles of these pathways in mediating the immunomodulatory activity of 1,25D. Furthermore, a subset of genes with evidence of interethnic differences in transcriptional response was also identified, suggesting that in addition to the well-established interethnic variation in circulating levels of vitamin D, the intensity of transcriptional response to 1,25D and LPS also varies between ethnic groups. We propose that dysregulation of the pathways identified in this study could contribute to immune-mediated disease risk.

Vitamin D plays an important immunomodulatory role through a transcriptional mechanism (Liu *et al.* 2006; Baeke *et al.* 2010; Aranow 2011; Hewison 2011). In the immune system, the active form of vitamin D, 1,25-dihydroxyvitamin D_3_ (1,25D), binds the vitamin D receptor (VDR), which translocates into the nucleus, where it modulates the transcription of genes with immune function, such as cathelicidin antimicrobial peptide (*CAMP*), defensin genes, such as β-defensin 4A (*DEFB4A*), and autophagy genes, such as autophagy related 5 (*ATG5*) (Liu *et al.* 2006, 2009; Adams *et al.* 2009; Yuk *et al.* 2009; Baeke *et al.* 2010; Aranow 2011; Hewison 2011). In monocytes/macrophages, 1,25D can be produced intracellularly from the inactive form, 25-hydroxyvitamin D_3_ (25D), which is found abundantly in circulation. The circulating levels of 25D vary greatly across individuals and ethnic groups (Rostand 2010; Looker *et al.* 2011; Murphy *et al.* 2012). Attesting to the important role of vitamin D in immune response, low levels of 25D have been linked to increased susceptibility to tuberculosis (Tb) (Nnoaham and Clarke 2008; White 2008). Moreover, 25D supplementation in individuals with hypovitaminosis D resulted in an enhanced antimicrobial response (Liu *et al.* 2006; Martineau *et al.* 2007; Adams *et al.* 2009). Although many studies have been conducted on the interindividual and interethnic variation in the circulating inactive 25D levels, with corresponding epidemiological links to immune-related diseases (Hypponen *et al.* 2001; Kamen *et al.* 2006; Kamen and Aranow 2008; Correale *et al.* 2009; Ascherio *et al.* 2010), little is known about interindividual and interethnic variation in the transcriptional response to active 1,25D.

Previous studies of 1,25D activity in immune cells highlight its complex immunomodulatory role, regulating activities such as enhancement of the response to *Mycobacterium tuberculosis* (*M. tb*) in THP-1 macrophage cell lines (Verway *et al.* 2013), downregulation of immune-related pathways such as interferon signaling in peripheral blood mononuclear cells (PBMCs) (Kupfer *et al.* 2013), and induction of a tolerogenic phenotype as well as an attenuation of the proinflammatory response in dendritic cells (van Halteren *et al.* 2004; Ferreira *et al.* 2012; Ferreira *et al.* 2015). Though the immunoregulatory role of 1,25D in different innate immune cell types is complex, it generally results in the attenuation of an intense proinflammatory response, which can have toxic consequences, such as sepsis and septic shock (Lehmann *et al.* 1987; Opal 2007; Zhang *et al.* 2012).

In this study, we focused on characterizing the transcriptional response to 1,25D in primary monocytes in the presence or absence of a proinflammatory stimulus, bacterial lipopolysaccharide (LPS). Stimulating monocytes with LPS enabled examination of how inflammation modifies the transcriptional response to 1,25D in monocytes. This analysis highlighted several biological pathways that are modulated by 1,25D in the absence of LPS (*e.g.*, oxidative phosphorylation and mitochondrial dysfunction), as well as others that are modulated by LPS and reversed by 1,25D (*e.g.*, proinflammatory cytokine signaling pathways). In addition, we identified some interethnic differential expression patterns, suggesting that the well-established interethnic variations in the vitamin D pathway extend to the intensity of transcriptional response to LPS and 1,25D.

## Materials and Methods

### Ethics statement

All donors to Research Blood Components (http://researchbloodcomponents.com/) and Sanguine Biosciences (https://www.sanguinebio.com/) sign an Institutional Review Board (IRB)-approved consent form giving permission to collect blood, and use it for research purposes. This study did not require IRB review at the University of Chicago because blood samples were not shipped with individually identifiable information.

### Subjects

All subjects were healthy donors collected by Research Blood Components and Sanguine Biosciences. Self-reported ethnicity, age, gender, date, and time of blood drawing were recorded for each donor. Buffy coats from 10 African-American (AA) and 10 European-American (EA) subjects were shipped within 24 hr of collection. We processed samples in multiple batches, balanced by ethnic group. Serum samples from the donors were sent to the Clinical Chemistry Laboratory of the University of Chicago to determine 25-hydroxyvitamin D_3_ (25D) levels and parathyroid hormone (PTH) levels. Total serum 25D and PTH levels were determined using electrochemiluminescence detection assays (cat. no. 06506780160 and cat. no. 11972103160, respectively, Roche Diagnostics Corporation, Indianapolis, IN).

### Monocyte culture and treatment

We isolated peripheral blood mononuclear cells (PBMCs) from the buffy coats of the 20 subjects by density gradient centrifugation using Ficoll-Paque PLUS medium (GE Healthcare Life Sciences, Pittsburgh, PA). We isolated monocytes from the PBMCs by positive selection using magnetic CD14 MicroBeads according to the supplier’s protocol (Miltenyi Biotec, San Diego, CA). We cultured isolated monocytes (1 × 10^6^ cells/mL) in RPMI 1640 medium (Gibco, Life Technologies, Grand Island, NY), 25 mg/mL Gentamicin (Gibco), and 10% charcoal-stripped fetal bovine serum (Gibco) in 24-well plates. Monocytes were cultured in three replicates for 24 hr for each of the following treatments: 1) vehicle solution containing 1% ethanol and 99% culture medium, as a negative control; 2) 100 nM of 1,25D; 3) 10 ng/mL of LPS in the vehicle solution; and 4) 100 nM of 1,25D and 10 ng/mL of LPS (experimental design summarized in Supplemental Material, Figure S1). These four treatments are abbreviated as E, V, L, and V + L, respectively.

### Transcriptome analysis

We pooled the three replicates for each treatment, and extracted total RNA from the pool using a Qiagen RNeasy Plus mini kit (Valencia, CA). We extracted RNA from 80 samples consisting of the 20 subjects that each received four treatments, in 10 batches each, balanced by ethnic group. RNA concentration and RNA integrity score (RIN) were recorded for each sample on a 2100 Bioanalyzer instrument (Agilent Technologies, Santa Clara, CA) (average RNA concentration and RIN scores in each ethnic group are summarized in Table S1). Total RNA was reverse transcribed into cDNA, labeled, hybridized to Illumina (San Diego, CA) Human HT-12 v3 Expression Beadchips, and scanned at the University of Chicago Functional Genomics Core facility. The microarrays were hybridized in three batches, and we recorded the array batch number for each sample to be used as a covariate in subsequent analyses. We performed low-level microarray analyses using the Bioconductor software package lumi (Du *et al.* 2008) in R, as previously described (Maranville *et al.* 2013). Briefly, we annotated probes by mapping their sequence to RefSeq (GRCh37) transcripts using BLAT. We discarded probes that mapped to multiple genes to avoid ambiguity in the source of a signal due to cross-hybridization of similar RNA molecules. We also discarded probes containing one or more HapMap SNPs to avoid spurious associations between expression measurements and ethnicity due to allele frequency differences between ethnic groups. We applied variance stabilization to all arrays, discarded poor quality probes, and quantile normalized the arrays using the default method implemented in the lumiN function. After these filters, probes mapping to 10,958 genes were used in downstream analyses (data available in File S1).

### Differential expression analysis

We tested each gene for differential expression (DE) using a linear mixed-effects model with the R package, lme4 (Bates *et al.* 2015). The model included fixed effects for ancestry, and the three treatment conditions (V, L, and V + L), as well as interaction effects between ancestry and the treatments. It also included a random effect to model the differences between the individuals. Lastly, the model included covariates for the technical factors with the strongest effects on the expression data (*P* < 0.05), as determined by their association with the principal components described below, including array batch, age, baseline 25D levels, baseline PTH levels, RNA concentration, and RIN scores. *P* values were obtained using the R package, lmerTest, which provides a summary function with *P* values added for the *t*-test based on the Satterthwaite approximation for denominator degrees of freedom (Kuznetsova *et al.* 2015). To correct for multiple testing, we estimated the false discovery rate (FDR) using the “qvalue” function in R, based on the Storey method (Storey and Tibshirani 2003). The FDR for DE was set at 1%. To identify genes that were DE between the two ancestries, we tested the significance of the fixed interaction effects between ancestry and the treatments. Here, we used a more relaxed FDR threshold of 10% to determine significance, due to the smaller sample size in the interethnic comparison (10 AA and 10 EA).

We also performed a joint Bayesian analysis using the R package Cormotif (Wei *et al.* 2015), which jointly models expression data across different experiments, enabling classification of genes into patterns of shared and distinct differential expression. Genes are assigned to correlation motifs, which are the main patterns of differential expression obtained from the shared information across experiments, which in our study are treatments and ethnic groups. We regressed out the technical covariates described above from the expression data using the limma package removeBatchEffect (Ritchie *et al.* 2015), and used the residuals as input. We used a modified version of Cormotif as described in Blischak *et al.* (2015), where the original code was modified to return the cluster likelihood for each gene to enable downstream analyses. Also, since Cormotif is nondeterministic, we ran each test 100 times and kept the result with the largest maximum likelihood estimate.

### Gene set enrichment analysis

We performed gene set enrichment analyses using the commercially available software Ingenuity Pathway Analysis (IPA). We compared DE genes with curated functional attribution lists organized by canonical pathway function. The magnitude of over-representation of a particular canonical pathway in the gene list from our study was calculated as the ratio of the number of genes from our data set that map to the pathway, divided by the total number of reference genes in that pathway in the IPA database. Statistical significance of the observed enrichment of a particular pathway was determined using Benjamini-Hochberg multiple testing corrected *P* values provided by IPA (Benjamini and Hochberg 1995).

### Identifying vitamin D receptor binding sites near DE genes

We reanalyzed published data sets of VDR ChIP-seq, which used THP-1 monocytic cell lines treated with 1,25D and LPS, or 1,25D alone (Tuoresmaki *et al.* 2014), and FAIRE-seq, which used THP-1 cells treated with 1,25D (Seuter *et al.* 2013). First, we aligned sequence reads to the human reference (GRCh37) using BWA backtrack 0.7.5 (Li and Durbin 2009). Second, we kept only sequence reads with phred-scaled mapping quality ≥ 30 using samtools v1.1 (Li *et al.* 2009). Third, PCR duplicates were removed with Picard v 1.130 (http://broadinstitute.github.io/picard/). For the ChIP-seq data sets, we confirmed the quality of data sets by strand cross-correlation (SCC) analysis (Landt *et al.* 2012) implemented in the R script “run_spp_nodups.R” packaged in phantompeakqualtools (https://code.google.com/p/phantompeakqualtools/). Statistically significant peaks were identified using MACS version 2 (Zhang *et al.* 2008) with the following essential command line arguments: macs2 callpeak–bw X -g hs–qvalue = 0.05 -m 5 50, where X is a length of the bandwidth that was defined as a fragment length calculated by SCC for the ChIP-seq data, or as 200 bp for the FAIRE-seq data reported in Seuter *et al.* (2013).

To identify VDR response elements, we considered peaks that overlapped completely or partially between the ChIP-seq data after 1,25D and LPS treatment and the FAIRE-seq data. We then annotated them using HOMER (Heinz *et al.* 2010) to find the closest gene to each peak, and, among these genes, we selected those that were DE genes in response to 1,25D (V) and the combined 1,25D + LPS (V + L) treatments from the linear mixed-effects analysis. Enrichment of VDR response elements was determined using Fisher’s exact test, comparing peaks in DE genes to those in nonDE genes.

We also examined the enrichment of VDR binding sites among genes clustered in each expression pattern from the joint Bayesian analysis using Cormotif. To inclusively identify VDR binding sites, we merged the ChIP-seq data from THP-1 cells treated with 1,25D and LPS treatment with data from THP-1 cells treated with 1,25D alone. We examined overlap between the genes that were closest to the peaks, and the genes in each Cormotif pattern. Enrichment of VDR peaks was then determined using Fisher’s exact test, comparing VDR peaks in each Cormotif pattern with peaks in the “No response” Cormotif pattern.

### Data availability

The raw microarray data files have been deposited in NCBI’s Gene Expression Omnibus (GEO) (Edgar *et al.* 2002), and are accessible through GEO Series accession number GSE78083 (http://www.ncbi.nlm.nih.gov/geo/query/acc.cgi?acc=GSE78083). The normalized expression values, the results from the linear mixed-effects model, and the results from Cormotif are provided in File S1.

## Results

### Sources of transcriptome-wide variation

To evaluate the variation in transcriptional response to 1,25D in the innate immune system in the presence and absence of an inflammatory stimulus, we cultured primary monocytes obtained from 10 African-American and 10 European-American healthy donors in four different conditions in parallel: i. EtOH (*i.e.*, the vehicle control, or E); ii. LPS (L); iii. 1,25 D (V); and iv. 1,25D plus LPS (V + L); the experimental design is illustrated in Figure S1. Transcript levels were measured with gene expression arrays for each treatment condition and each individual, resulting in a total of 80 transcriptome data sets. Relevant covariates, including serum levels of 25D, were measured or recorded and used in downstream analyses (see *Materials and Methods*). Although there was significant interindividual and interethnic variation in serum 25D levels in our sample of donors (Table S1), this variable was not correlated with the transcriptional response to LPS, 1,25D, or their combination (Figure S5). This suggests that our *in vitro* system is not affected by 25D levels *in vivo*.

To evaluate the sources of variation in the overall transcriptome data, we performed principal components analysis (PCA) of the variance-stabilized log_2_-transformed expression data using the prcomp function in R. Principal component 1 (PC1) separates the samples by LPS treatment, accounting for 22% of the total variation in gene expression, and reflecting the large effect of LPS on the transcriptome (Figure S2, A and C, and Table S2), while PC2 separates the samples by 1,25D treatment, and accounts for 8.6% of the total variation in gene expression (Figure S2, A and D, and Table S2). PC3 and PC4, which account for 6.7% and 5.8% of variation respectively, separate the samples by the three array processing batches (Figure S2, B, E, and F, and Table S2). We also tested for associations between the PCs and the different covariates recorded for each sample (average sample covariates are compared between ancestries in Table S1). PC1 was associated with RNA concentration (*P* = 1.54 × 10^−5^, *r*^2^ = 0.219), PC2 was weakly associated with the RNA integrity number (RIN) scores (*P* = 9.0 × 10^−3^, *r*^2^ = 0.086), while PC3 was associated with age (*P* = 1.36 × 10^−6^, *r*^2^ = 0.266), baseline 25D levels (*P* = 1.0 × 10^−3^, *r*^2^ = 0.136), baseline PTH levels (*P* = 6.1 × 10^−6^, *r*^2^ = 0.237), and RIN score (*P* = 5.0 × 10^−3^, *r*^2^ = 0.099) (Table S2). The effects of array processing batch, RNA concentration, RIN scores, serum 25D, and PTH levels were subsequently included as covariates in the linear mixed-effects model for differential expression.

After regressing out the covariates using the limma package removeBatchEffect, and performing PCA on the residuals of the covariates-corrected expression data, we observed that PC1 and PC2 separated the samples by treatment (Figure S3, A, C, and D, and Table S3), but PC1 was still associated with RNA concentration (*P* = 3.56 × 10^−5^, *r*^2^ = 0.203) while PC2 was still associated with RIN score (*P* = 3 × 10^−3^, *r*^2^ = 0.113) (Table S3). PC3, which accounted for 5% of the total variation in gene expression, was associated with sample (*P* = 4.5 × 10^−7^, *r*^2^ = 0.286), and ancestry (*P* = 1.04 × 10^−5^, *r*^2^ = 0.227), highlighting the effect of interindividual and interethnic variation on gene expression (Figure S4 and Table S3).

### Opposite effects of 1,25D and LPS on the transcriptome

Using the main effects for each treatment from the linear mixed-effects model, we identified genes that were DE in response to the different treatment conditions at a FDR of 1%. A total of 2888 genes were DE in response to 1,25D alone relative to vehicle (V *vs.* E). Gene set enrichment analysis identified metabolic processes, such as oxidative phosphorylation and the tricarboxylic acid (TCA) cycle, enriched among upregulated genes (Table S4). Pathways that play important roles in regulating translation processes, such as EIF2 signaling and mTOR signaling, were also significantly enriched among upregulated genes, indicating an important role of 1,25D in regulating translation. Immune responses involving chemokine signaling, B and T cell signaling, as well as various proinflammatory signaling cascades, such as Tec kinase signaling, phospholipase C signaling, and integrin signaling, were enriched among the downregulated genes, consistent with the immunomodulatory function of 1,25D.

There was a strong transcriptomic response to LPS treatment relative to vehicle (L *vs.* E), with 4461 genes DE at a FDR of 1%. Pathways enriched among LPS responsive genes highlight the opposite direction of transcriptional response to 1,25D and LPS, where proinflammatory immune response pathways were enriched among upregulated genes, while oxidative phosphorylation and translational control pathways were enriched among downregulated genes (Table S4), indicating the importance of these pathways in the proinflammatory effects induced by LPS stimulation.

### Effects of combined 1,25D + LPS treatment on the transcriptome

The combined treatment of 1,25D + LPS resulted in 4720 genes significantly DE relative to vehicle (V + L *vs.* E). We also examined the transcriptional response of the combined 1,25D + LPS treatment relative to LPS (V + L *vs.* L) in an attempt to isolate the effect of 1,25D on the transcriptome in the presence of LPS, and identified 2404 genes significantly DE in this treatment category.

The pattern of response to V + L *vs.* E followed a similar pattern to LPS treatment alone (L *vs.* E), with similar pathways enriched among genes in these treatment categories (Table S4), probably because of the overwhelming transcriptional response to LPS. Genes significantly DE in response to V + L *vs.* L, which effectively subtracts the transcriptional effects of LPS, were similar to the genes significantly DE in response to 1,25D treatment alone (V *vs.* E), with similar pathways enriched.

We detected additional pathways enriched among genes significantly DE in response to the combined V + L treatments, both relative to vehicle and relative to LPS. These included adipogenesis and insulin receptor signaling pathways, both involved in lipid metabolic processes, which were enriched among upregulated genes. IL-4 signaling, which is associated with allergy and asthma through development of T-cell-mediated immune responses (Chatila 2004; LaPorte *et al.* 2008), was significantly enriched among downregulated genes (Table S4). Pathways enriched among genes responsive to the combined V + L treatment indicate a regulatory role of 1,25D in these pathways specifically in the context of LPS stimulation.

### Bayesian analysis of shared transcriptional response across treatments and ethnic groups

**Figure 1:**
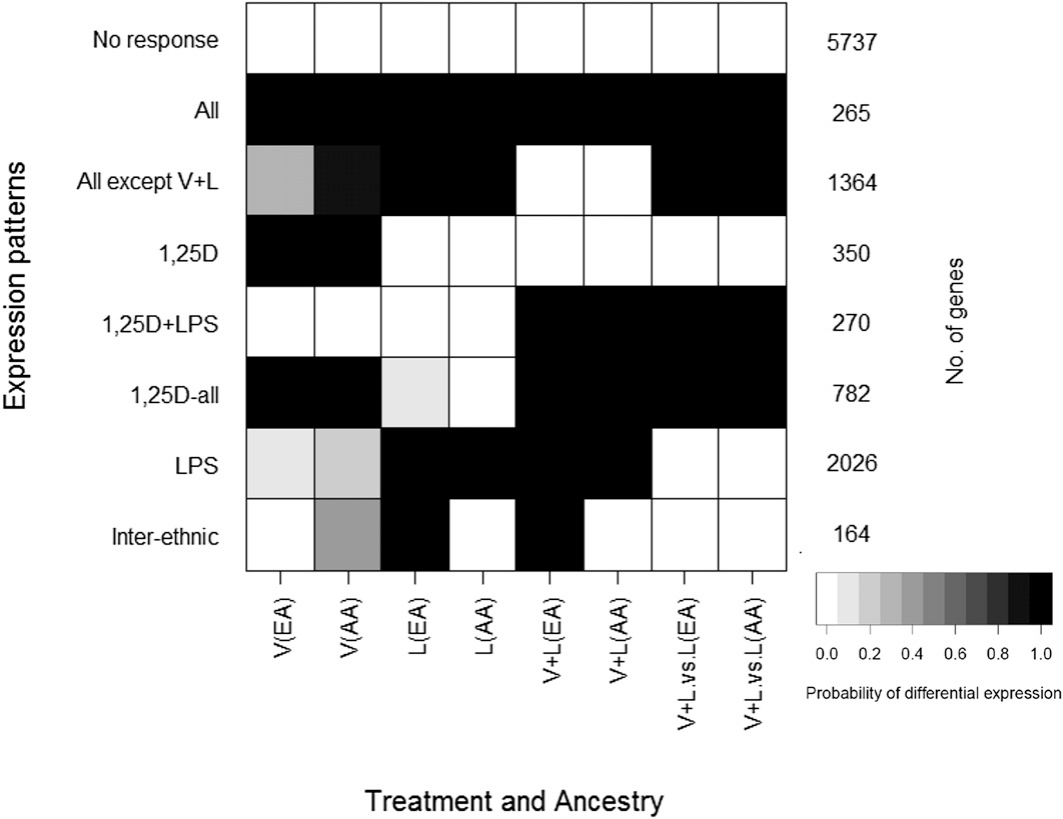
Transcriptional response patterns shared across the different treatments and ancestries identified by implementing a joint Bayesian analysis using Cormotif. The shading of each box represents the posterior probability that a gene assigned to a given expression pattern (rows) is differentially expressed in individuals from a particular ancestry in response to each treatment (columns). V, response to 1,25D, relative to vehicle; L, response to LPS relative to vehicle; V + L, response to 1,25D + LPS relative to vehicle; V + L.*vs.*L, response to 1,25D+LPS relative to LPS; EA, European-American; AA, African-American.

**Figure 2:**
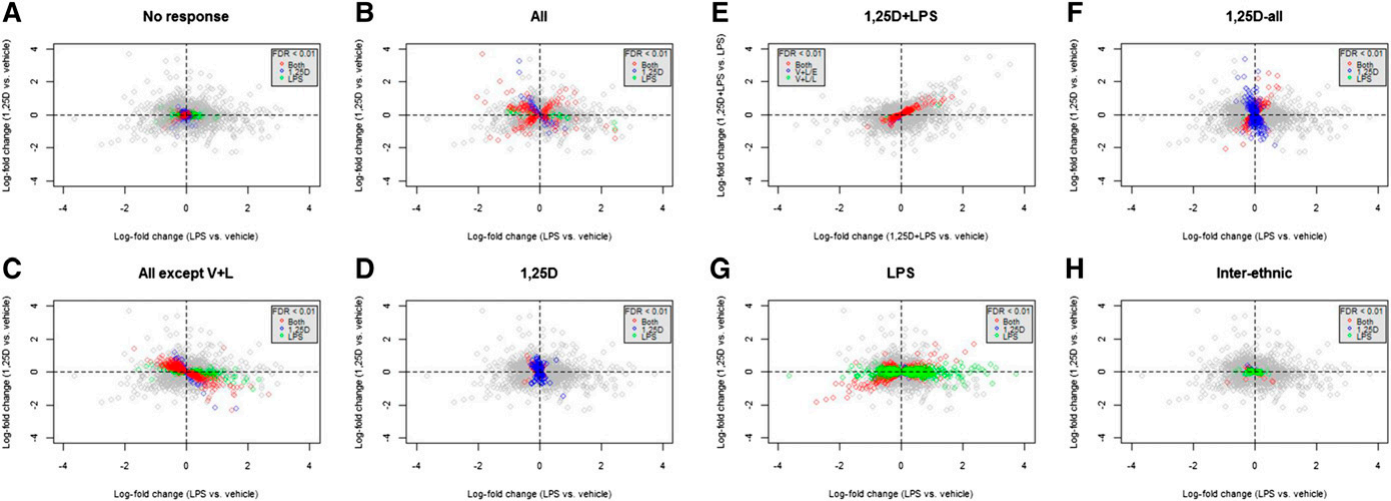
Direction of response in the different correlation motifs. Patterns of differential response to single treatment with 1,25D (vertical axis) or LPS (horizontal axis) for each correlation motif are shown in (A)–(D) and (F)–(H). (E) Patterns of differential response to the combined treatment with 1,25D and LPS relative to LPS (vertical axis), and 1,25D and LPS relative to vehicle (horizontal axis). Genes are color coded based on q-values < 0.01 from linear mixed-effects analysis as follows: red, DE in response to both 1,25D and LPS; blue, DE in response to 1,25D; green, DE in response to LPS; gray, not DE.

To further dissect the effects of 1,25D and LPS on the transcriptome, we sought to identify the shared and distinct patterns of transcriptional response across treatments, and across ethnic groups. A popular approach to this question is to investigate the overlap of DE genes between conditions at a given FDR threshold. However, this approach fails to account for incomplete power to detect DE genes, thus exaggerating the differences in the transcriptional response between the conditions. In order to identify shared patterns of transcriptional response across treatments and ancestry while accounting for incomplete power, we implemented a joint Bayesian analysis with the R/Bioconductor package Cormotif (Wei *et al.* 2015). Genes were classified into different response patterns, or correlation motifs, across treatments and ancestry (Figure 1). Since Cormotif does not distinguish the direction of effect across treatments, we used the results of the linear mixed-effects model in conjunction with the Cormotif approach to establish direction of response in the different response patterns (Figure 2).

A total of 5737 genes were classified in the “No response” pattern, which includes genes whose expression levels were unchanged across all the treatments (Figure 1 and Figure 2A). This is broadly consistent with the results of the linear mixed-effects model, with 80% of these genes being also classified as nonDE for any treatment in the linear mixed-effects model.

**Figure 3:**
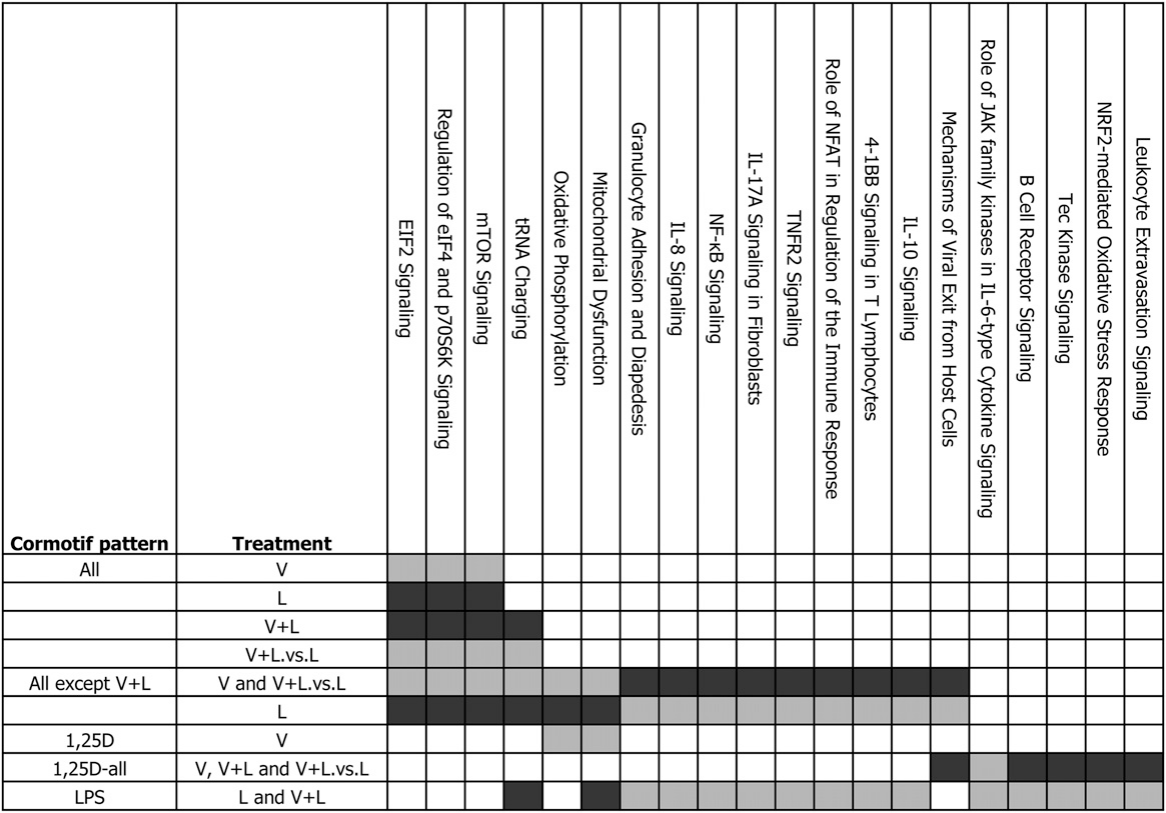
Sharing of enriched biological pathways across Cormotifs. The table shows the biological pathways that were enriched (FDR < 0.05) in more than one Cormotif subdivided based on the direction of transcriptional response (upregulated genes in light gray, and downregulated genes in dark gray), and the treatment (V, response to 1,25D, relative to vehicle; L, response to LPS relative to vehicle; V + L, response to 1,25D + LPS relative to vehicle; V + L.*vs.*L, response to 1,25D + LPS relative to LPS).

Genes that responded to all the treatments were classified in the “All” pattern, and included 265 genes whose expression levels changed across all treatments and ancestries (Figure 1 and Figure 2B). Genes classified in this Cormotif had response patterns to 1,25D and LPS that were both concordant (*i.e.*, up or downregulated in both treatments) and discordant (*i.e.*, upregulated in one treatment and downregulated in the other). Genes that were upregulated in all treatments (top-right quadrant, Figure 2B) included *CD14*, which encodes a surface antigen expressed on monocytes that is involved in mediating response to bacterial LPS. Genes that were downregulated in all treatments included chemokine signaling genes such as *CCL13* (bottom-left quadrant, Figure 2B). The discordant response patterns included genes that were upregulated by 1,25D and downregulated by LPS (top-left quadrant, Figure 2B), with EIF2 signaling and mTOR signaling pathways significantly enriched among these genes (Figure 3). This is consistent with the opposite transcriptional effects of 1,25D and LPS on genes in these pathways that were highlighted in the linear mixed-effects analysis. Genes that were downregulated by 1,25D and upregulated by LPS (bottom-right quadrant, Figure 2B) included some cytokine receptor genes such as *IL7R* and *IL2RA*, which are important components of the proinflammatory signaling cascade.

The “All except V + L” pattern included 1364 genes whose expression levels changed in all treatments, except the combined 1,25D + LPS relative to vehicle (V + L *vs.* E). All the genes in this Cormotif pattern were discordant in their response to 1,25D and LPS, resulting in a neutral effect in the response to the combined V + L *vs.* E (Figure 1 and Figure 2C). Genes that were responsive to the combined V + L *vs.* L followed a similar direction of response to the genes DE in response to the individual V *vs.* E treatment, suggesting that the response to 1,25D at these genes is not dramatically influenced by LPS. The genes that were upregulated by 1,25D and downregulated by LPS (top-left quadrant, Figure 2C) were enriched for EIF2 and mTOR signaling pathways, similar to the discordant genes in the “All” category (Figure 3). In addition, oxidative phosphorylation and mitochondrial dysfunction pathways were significantly enriched among these genes. On the other hand, genes that were downregulated by 1,25D and upregulated by LPS (bottom-right quadrant, Figure 2C) were enriched for various proinflammatory response pathways, including granulocyte adhesion and diapedesis, IL-8 signaling, NF-kB signaling, TNFR2 signaling, and Role of NFAT in regulation of the immune response (Figure 3).

Genes responsive to 1,25D were divided into three Cormotif patterns: “1,25D”, “1,25D + LPS”, and “1,25D-all”. The “1,25D” pattern included 350 genes that were DE in response to V *vs.* E alone (Figure 1 and Figure 2D). Oxidative phosphorylation and mitochondrial dysfunction pathways were significantly enriched among upregulated genes (Figure 3). Interestingly, the oxidative phosphorylation pathway genes enriched in this Cormotif pattern responded similarly to the genes in the same pathway classified in the “All except V + L” Cormotif pattern, in that they are significantly induced by 1,25D. However, the oxidative phosphorylation pathway genes in the “1,25D” pattern respond exclusively to 1,25D, while those in the “All except V + L” Cormotif pattern are upregulated by 1,25D, and downregulated by LPS (Figure 3 and Figure S6). This indicates a context-specific response profile among genes in the same pathway, where some genes in the oxidative phosphorylation pathway are uniquely regulated by 1,25D, whereas other genes in the same pathway are regulated by both 1,25D and LPS.

The “1,25D+LPS” pattern included 270 genes that responded to 1,25D only in the presence of LPS. This pattern captured genes that were DE in response to the combined V + L *vs.* E, and V + L *vs.* L (Figure 1 and Figure 2E). Although there were no enriched pathways among genes in this Cormotif pattern at an FDR of 5%, some interesting pathways, such as eNOS signaling and cholesterol biosynthesis pathway, were represented among the downregulated genes at a FDR of 27%, suggesting a role for 1,25D in modulating these pathways upon LPS stimulation.

The “1,25D-all” pattern included 782 genes that were DE in response to 1,25D in the both the presence and absence of LPS (Figure 1 and Figure 2F). Genes in the antimicrobial pathway were included in this category, such as the antibacterial peptide gene *CAMP*, autophagy genes *ATG3*, *ATG5*, *ATG2A*, and *ATG9A*, and the intracellular pattern recognition receptor gene *NOD2*. These genes were significantly upregulated in response to 1,25D, alone or in combination with LPS. The role of JAK family kinases in IL-6-type cytokine signaling was the most significantly enriched pathway among the upregulated genes (Figure 3), and included genes such as *MAPK14*, *PTPN11*, and *STAT5B*, all of which could be crucial for triggering antimicrobial responses in monocytes. Biological pathways enriched among downregulated genes in this category included B cell receptor signaling, Tec kinase signaling and leukocyte extravasation signaling (Figure 3 and Figure S7), highlighting the role of 1,25D in repressing proinflammatory response pathways. Interestingly, immunological and inflammatory diseases were among the most enriched disease categories from the IPA analysis among the downregulated genes (Table S6 and Figure S7), suggesting a protective role of 1,25D in immunological diseases. Overall, the “1,25D-all” response pattern illustrates the important dual immunomodulatory role played by 1,25D in monocytes, where antimicrobial pathway genes are upregulated, while proinflammatory pathway genes associated with immunological and inflammatory disease are downregulated by 1,25D in the presence or absence of LPS stimulation.

The “LPS” pattern included 1400 genes whose expression levels changed in response to L *vs.* E and the combined V + L *vs.* E (Figure 1 and Figure 2G). Consistent with the results from the linear mixed-effects model, proinflammatory pathways were significantly enriched among the upregulated genes in this category, including IL-8 signaling, NF-kB signaling, IL-17 signaling, and TNFR2 signaling, among others (Figure 3). Among the downregulated genes, tRNA charging, mitochondrial dysfunction, the TCA cycle II, galactose metabolism pathway, and folate transformation pathway were significantly enriched (Figure 3 and Table S5), indicating that LPS modulates transcription of genes in these metabolic pathways.

### Genes with interethnic differential response

The “interethnic” pattern was of particular interest, as it identified 164 genes with evidence of differential responses to LPS treatments between AA and EA subjects, with a stronger response in EA compared to AA (Figure 1 and Figure 2H).

**Figure 4:**
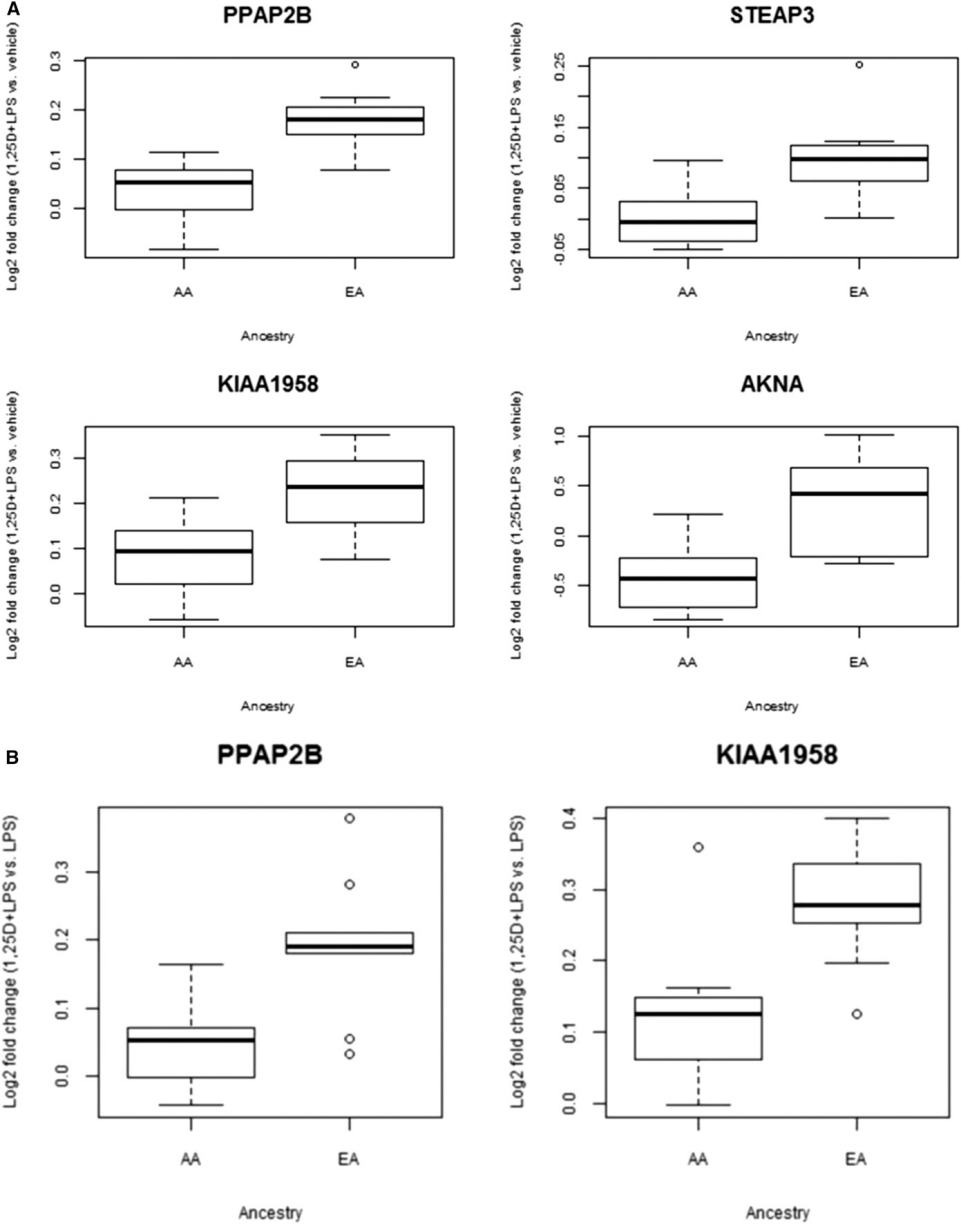
Interethnic differential response patterns. Genes with interethnic differences in transcriptional responses to 1,25D + LPS relative to vehicle (A), and relative to LPS (B) were identified using the interaction term for treatment and ancestry in the linear mixed-effects model (FDR < 0.10). The boxplots show examples of these genes with different log-fold change in transcript levels between the two ethnic groups. AA, African-American; EA, European-American.

We also interrogated the degree of interethnic differences in transcriptional response using the main interaction term for treatment and ancestry in the linear mixed-effects model. We identified 15 genes with strong interethnic differences in response to V + L *vs.* E at a FDR < 10%. These genes include *PPAP2B*, which encodes a member of the phosphatidic acid phosphatase (PAP) family and has been implicated in coronary artery disease risk (Schunkert *et al.* 2011; Dichgans *et al.* 2014); *STEAP3*, which encodes an endosomal ferrireductase required for efficient transferrin-dependent iron uptake; and *AKNA*, which encodes a transcription factor that specifically activates the expression of the CD40 receptor and its ligand CD40L/CD154 on lymphocyte cell surfaces, which are critical for antigen-dependent-B-cell development (Figure 4A). Interestingly, 13 out of the 15 genes showed more significant differential responses in EA (Figure S8), similar to the pattern observed in the “interethnic” Cormotif pattern.

To account for the effect of LPS, and examine the extent to which the interethnic differential response patterns were modulated by 1,25D, we examined interethnic differential response to the combined V + L *vs.* L. *PPAP2B* and *KIAA1958* were the only statistically significant genes identified in this category (*P* = 2.96 × 10^−6^ and 9.62 × 10^−6^, respectively), with both of these genes more significantly differentially expressed in EA (Figure 4B).

### Regulatory elements near DE genes

We examined the overlap between genes DE in response to 1,25D and the combined 1,25D + LPS treatments in our primary monocytes, and published datasets for VDR ChIP-seq (Tuoresmaki *et al.* 2014) and FAIRE-seq (Seuter *et al.* 2013) performed in THP-1 monocytic cells, to examine whether there was enrichment of open chromatin regions and VDR binding sites near the transcription start sites of DE genes. We found a significant enrichment of VDR binding sites among genes DE in response to V *vs.* E (*P* = 4.56 × 10^−11^), V + L *vs.* E (*P* = 3.97 × 10^−8^), and V + L *vs.* L (*P* = 1.54 × 10^−7^) (Table S7). There was an overlap of 201 genes between the DE genes, VDR ChIP-seq, and FAIRE-seq datasets, highlighting genes such as *CAMP* and *CD14*, which contain open chromatin regions and VDR binding sites near the transcription start site; these 201 genes are potentially direct VDR targets.

In addition, we examined the enrichment of VDR binding sites across the different Cormotif patterns (Table 1). The genes in the “1,25D-all” and “All” Cormotif patterns had the highest enrichment of VDR binding sites (*P* = 6.88 × 10^−13^ and 2.57 × 10^−8^, respectively), indicating a higher proportion of potentially direct VDR targets represented in these Cormotif patterns. Genes in the “1,25D” Cormotif pattern were not significantly enriched for VDR binding sites, suggesting that the presence of LPS, in addition to 1,25D, is important to enable the 1,25D-VDR transcriptional activity in primary monocytes.

**Table 1:** Proportion of genes in each Cormotif pattern containing vitamin D receptor (VDR) binding sites

Cormotif Pattern	Total No. Genes	No. Genes with VDR Binding Site	Proportion of Genes with VDR Binding Site	Enrichment *P* Value
No response	5737	186	0.03	—
All	265	31	0.12	2.57 × 10^−8^
All except V + L	1364	65	0.05	0.01
1,25D	350	17	0.05	0.13
1,25D + LPS	270	23	0.09	1.49 × 10^−4^
1,25D-all	782	76	0.10	6.88 × 10^−13^
LPS	2026	96	0.05	3.79 × 10^−3^
Interethnic	164	8	0.05	0.27

Enrichment of VDR peaks in each category was calculated using Fisher’s exact test, comparing genes in each Cormotif pattern to those in the “No response” Cormotif pattern.

## Discussion

We used a transcriptomic approach to characterize the immunomodulatory role of 1,25D in the presence of a proinflammatory stimulus to identify the mechanisms through which 1,25D exerts its immunomodulatory role. We analyzed differential expression patterns using both a linear mixed-effects analysis, which modeled individual treatment comparisons, and a Bayesian analysis using the Cormotif method, which jointly modeled differential expression across all treatments and ethnic groups, thereby accounting for incomplete power. A similar joint Bayesian framework has been successfully applied to expression quantitative trait loci (eQTL) mapping to distinguish between shared and context-specific eQTLs (Maranville *et al.* 2011; Flutre *et al.* 2013). Our joint Bayesian analysis enabled clustering of DE genes into distinct transcriptional response patterns, with pathways enriched within these transcriptional patterns highlighting mechanisms that mediate the immunomodulatory role of 1,25D.

Metabolic pathways involving oxidative phosphorylation were enriched among upregulated genes in the “All except V+L” and “1,25D” Cormotif patterns (Figure 3). We highlight context-specific response pattern of genes within this pathway, where some genes were uniquely induced by 1,25D, while genes in other parts of the pathway were regulated by both 1,25D and LPS. The crucial role played by 1,25D in regulating oxidative phosphorylation was previously reported in PBMCs and dendritic cells (Ferreira *et al.* 2012, 2015; Kupfer *et al.* 2013), and this regulation of metabolic reprogramming by 1,25D is thought to be crucial for controlling function, growth, proliferation, and survival of various immune cell subsets (Williams *et al.* 2013; Kelly and O’Neill 2015). The fact that LPS downregulated genes in the oxidative phosphorylation pathway confirms previous reports indicating that LPS induces a metabolic shift away from oxidative phosphorylation to anaerobic glycolysis in macrophages and dendritic cells to enable ATP production (Kelly and O’Neill 2015). This effect is similar to the Warbug effect in tumor cells, whose high energy demand is met by switching the metabolic profile away from the TCA cycle and the oxidative phosphorylation pathway toward glycolysis, thereby enabling rapid ATP production (Warburg *et al.* 1927; Kelly and O’Neill 2015). Previous work done in mouse macrophages and dendritic cells (Krawczyk *et al.* 2010; Freemerman *et al.* 2014) indicated that a metabolic shift toward glycolysis mediated the proinflammatory response, and this proinflammatory response could be attenuated by pharmacologic inhibition of glycolysis. From our study, this subset of oxidative phosphorylation pathway genes that were downregulated by LPS, were then upregulated by addition of 1,25D in combination with LPS (Figure 3). Therefore, oxidative phosphorylation could be one of the mechanisms through which 1,25D attenuates the proinflammatory response induced by LPS in monocytes, and the subset of genes in this pathway that we identified which were modulated by both LPS and 1,25D could be central to this mechanism.

The mTOR signaling pathway was consistently enriched among genes that were upregulated by 1,25D and downregulated by LPS in the “All” and “All except V + L” patterns. mTOR signaling was previously implicated in inhibition of proinflammatory response in LPS-stimulated monocytes/macrophages and dendritic cells, as well as in the maintenance of a tolerogenic phenotype in dendritic cells (Ohtani *et al.* 2008; Weichhart *et al.* 2008; Wang *et al.* 2011; Ferreira *et al.* 2015). Inhibition of mTOR resulted in increased proinflammatory cytokine production by LPS-stimulated monocytes/macrophages and dendritic cells (Ohtani *et al.* 2008; Weichhart *et al.* 2008), and increased T cell proliferation (Ohtani *et al.* 2008; Ferreira *et al.* 2015), implicating a role of mTOR in regulating the proinflammatory response. The genes in this pathway were significantly downregulated by LPS; however, this direction of response was reversed by addition of 1,25D in combination with LPS (Figure 3), implying that 1,25D attenuates the proinflammatory response by upregulating mTOR signaling. In addition, the genes in this pathway play important roles in regulating translation initiation, and include the ribosomal protein gene *RPS27*, and the eukaryotic translation initiation factor gene *EIF2A*, which encodes the eukaryotic initiation factor 2 (eIF-2α), which has been shown to be a downstream target of the vitamin D receptor (Wu *et al.* 2010). Therefore, regulation of translation initiation through targeting the mTOR signaling pathway could be a novel mechanism for the attenuation of the proinflammatory response mediated by 1,25D in monocytes.

Furthermore, the EIF2 signaling pathway was also enriched among genes upregulated by 1,25D and downregulated by LPS in the “All” and “All except V + L” patterns, and this result is consistent with the individual treatment DE analysis using the linear mixed-effects model (Table 1, Table S4, and Table S5). EIF2 signaling plays an important role in regulating translation initiation in response to stress, and was implicated in regulating proinflammatory cytokine production and bacterial invasion in mouse embryonic fibroblast cells (MEFs) (Shrestha *et al.* 2012). Shrestha *et al.* (2012) reported that the *Yersinia*-encoded virulence factor, YopJ, inhibited EIF2 signaling in MEFs. Similarly, in our study, LPS consistently downregulated genes in the EIF2 signaling pathway, in a mechanism that might be similar to that triggered by YopJ. In addition, Shrestha *et al.* (2012) observed that mutant MEFs with defective EIF2 signaling that were infected with different bacterial pathogens experienced enhanced cytotoxicity compared to wild type, due to increased bacterial invasion, indicating a direct role for EIF2 signaling in the antimicrobial response; 1,25D could hence exert its antimicrobial role in monocytes by upregulating genes in the EIF2 signaling pathway.

The dual immunomodulatory role of 1,25D was also highlighted by the genes clustered in the “1,25D-all” pattern. While 1,25D broadly downregulated genes in the proinflammatory cytokine and signaling cascade pathways, it also played a crucial role in inducing important antimicrobial and autophagy genes in this Cormotif pattern. The most significantly enriched biological pathway among the upregulated genes was the role of JAK family kinases in IL-6-type cytokine signaling, which contained genes such as *STAT5B*, which regulates signaling in diverse biological processes. Previous reports indicate that the TLR2/1-mediated induction of the vitamin D-dependent antimicrobial pathway requires IL-15 activity (Krutzik *et al.* 2008), which could be mediated via STAT5 activation, which has been shown to be important for IL-15 signaling (Musikacharoen *et al.* 2001; Pandiyan *et al.* 2012). Hence, 1,25D could regulate genes in this pathway to trigger antimicrobial responses in monocytes.

By profiling transcriptional response in monocytes from individuals of African-American and European-American ancestries, we identified some patterns of interethnic variation in response to LPS, and the combined 1,25D + LPS treatment in both the linear mixed-effects analysis and the joint Bayesian analysis, while correcting for interindividual variation in baseline levels of circulating 25D. This raises the intriguing possibility that interethnic variation in the vitamin D pathway is not limited to the well-established differences in circulating levels of 25D (Rostand 2010; Looker *et al.* 2011; Murphy *et al.* 2012), but it may extend to the intensity of the transcriptional response to LPS and 1,25D. Interestingly, most of the genes with interethnic differential response showed more significant differential responses in EA. The fact that most of the interethnic transcriptional differences were detected in the response to LPS or to the combined 1,25D + LPS, both relative to vehicle, suggests that these two ethnic groups differ in the proinflammatory transcriptional response. However, two genes had significant interethnic differences in transcriptional response to the combined 1,25D + LPS relative to LPS (Figure 4B), suggesting that they differ more specifically in their response to vitamin D.

We further identified enrichment of VDR ChIP-seq and FAIRE-seq peaks among genes DE in response to the combined 1,25D + LPS treatments. This enrichment was particularly strong for genes in the “1,25D-all” and “All” Cormotif patterns, suggesting that a substantial proportion of these genes are under direct regulation of the 1,25D-VDR transcription factor complex. Intriguingly, we did not detect an enrichment of VDR binding sites near genes in the “1,25D” Cormotif. Different explanations could account for this observation. One is that the combination of both 1,25D and LPS is important for stimulating the transcriptional activity of the 1,25D-VDR transcriptional complex in human monocytes (Liu *et al.* 2006; Adams *et al.* 2009; Tuoresmaki *et al.* 2014). On the other hand, because the genes in the “1,25D” Cormotif are observed to respond only to one treatment condition, it is possible that they are enriched for false positives relative to genes in other Cormotifs that are found to respond to multiple treatment conditions. Another caveat to this analysis is that we examined the overlap of VDR ChIP-seq peaks from published data sets with experimental conditions that were different to ours. While we treated primary monocytes with 100 nM 1,25D and 10 ng/mL LPS for 24 hr, the VDR ChIP-seq data were obtained from THP-1 monocytic cell lines cultured with 100 ng/mL LPS for 24 hr, and then treated with 10 nM 1,25D for 80 min (Tuoresmaki *et al.* 2014). Future VDR ChIP-seq studies with uniform experimental conditions in primary monocytes will enable better characterization of the regulatory architecture of 1,25D response genes.

Overall, through transcriptomic profiling, our study characterizes the dual immunomodulatory role of 1,25D in primary human monocytes, highlighting the importance of biological pathways such as mTOR signaling and EIF2 signaling in mediating this immunomodulatory role. The pathways highlighted in this study may provide mechanistic clues for the observed associations between insufficient levels of circulating serum 25D and increased disease risk. The interindividual and interethnic variation in intracellular transcriptional response to 1,25D has not been previously characterized, and could serve as an additional contribution to disease risk.

## Supplementary Material

Supplemental Material
