# Supplementary material for: Patterns of Transcriptional Response to 1,25-Dihydroxyvitamin D3 and Bacterial Lipopolysaccharide in Primary Human Monocytes
Source: G3 (Bethesda). 2016 Mar 11;6(5):1345–55. doi: 10.1534/g3.116.028712 (PMC4856085; doi:10.1534/g3.116.028712)
Supplement: Supplemental Material [file supp_6_5_1345__index.html]

Patterns of Transcriptional Response to 1,25-Dihydroxyvitamin D3 and Bacterial Lipopolysaccharide in Primary Human Monocytes — Supplemental Material 

# Patterns of Transcriptional Response to 1,25-Dihydroxyvitamin D3 and Bacterial Lipopolysaccharide in Primary Human Monocytes

## Supplemental Material for Kariuki *et al.*, 2016

**Files in this Data Supplement:**

- Figure S1 - Experimental Design: Primary monocytes were isolated from peripheral blood mononuclear cells (PBMCs) obtained from twenty healthy individuals of African-American (AA) and European-American (EA) ancestry. (.pdf, 68 KB)
- Table S2 - Principal components analysis of variance-stabilized log2-transformed expression data. (.pdf, 27 KB)
- Table S3 - Principal components analysis of variance-stabilized log2-transformed expression data adjusted for covariates. (.pdf, 44 KB)
- Table S4 - Biological pathways enriched at a FDR < 0.05 among genes significantly DE in response to single treatment with 1,25D or LPS, identified using linear mixed-effects model. (.pdf, 42 KB)
- Table S5 - Biological pathways enriched at a FDR < 0.05 among genes in the different Cormotif patterns. (.pdf, 100 KB)
- Table S6 - Diseases enriched among down-regulated genes in the "1,25D-all" Cormotif pattern at a FDR < 0.05. B-H p-value\* = Benjamini-Hochberg multiple testing corrected p-value. (.pdf, 26 KB)
- Table S7 - Enrichment of VDR ChIP-seq peaks among genes responsive to 1,25D treatment. (.pdf, 19 KB)
- Figure S2 - Principal components analysis (PCA) of the expression data indicating the sources of transcriptome-wide variation. (.pdf, 50 KB)
- Figure S3 - Principal components analysis (PCA) of covariates-corrected expression data indicating the sources of transcriptome-wide variation after correction for technical covariates. (.pdf, 89 KB)
- Figure S4 - Boxplot showing inter-ethnic variation in covariates-corrected expression data captured by PC3 and PC4, with the proportion of variation explained in parenthesis. (.pdf, 25 KB)
- Figure S5 - Examining the effect of baseline 25D levels on transcriptional response. (.pdf, 171 KB)
- Figure S6 - Boxplots of genes in the Oxidative Phosphorylation pathway clustered in the "1,25D" and "All except V+L" Cormotif patterns with treatment-specific response patterns. (.pdf, 68 KB)
- Figure S7 - Network of down-regulated genes in the "1,25D-all" Cormotif pattern. Biological pathways enriched among these genes are highlighted in cyan. (.pdf, 634 KB)
- Figure S8 - Boxplots of genes with different log fold change in transcript levels between the two ethnic groups in response to 1,25D+LPS relative to vehicle (V + L vs. E) at a FDR < 0.10. 13 genes showed stronger response in EA's, while the 2 genes indicated with asterisks (*AKNA*, and *DEGS1*) showed stronger response in AA's. (.pdf, 44 KB)
- Table S1 - Comparing averaged sample covariates data across ancestries, with p-values obtained from t-test. (.pdf, 14 KB)
- File S1 - The normalized expression values, the results from the linear mixed-effects model, and the results from Cormotif. (.xlsx, 17 MB)
