# Supplementary material for: Patterns of Transcriptional Response to 1,25-Dihydroxyvitamin D3 and Bacterial Lipopolysaccharide in Primary Human Monocytes
Source: G3 (Bethesda). 2016 Mar 11;6(5):1345–55. doi: 10.1534/g3.116.028712 (PMC4856085; doi:10.1534/g3.116.028712)
Supplement: Supplemental Material [file supp_g3.116.028712_FigureS1.pdf]

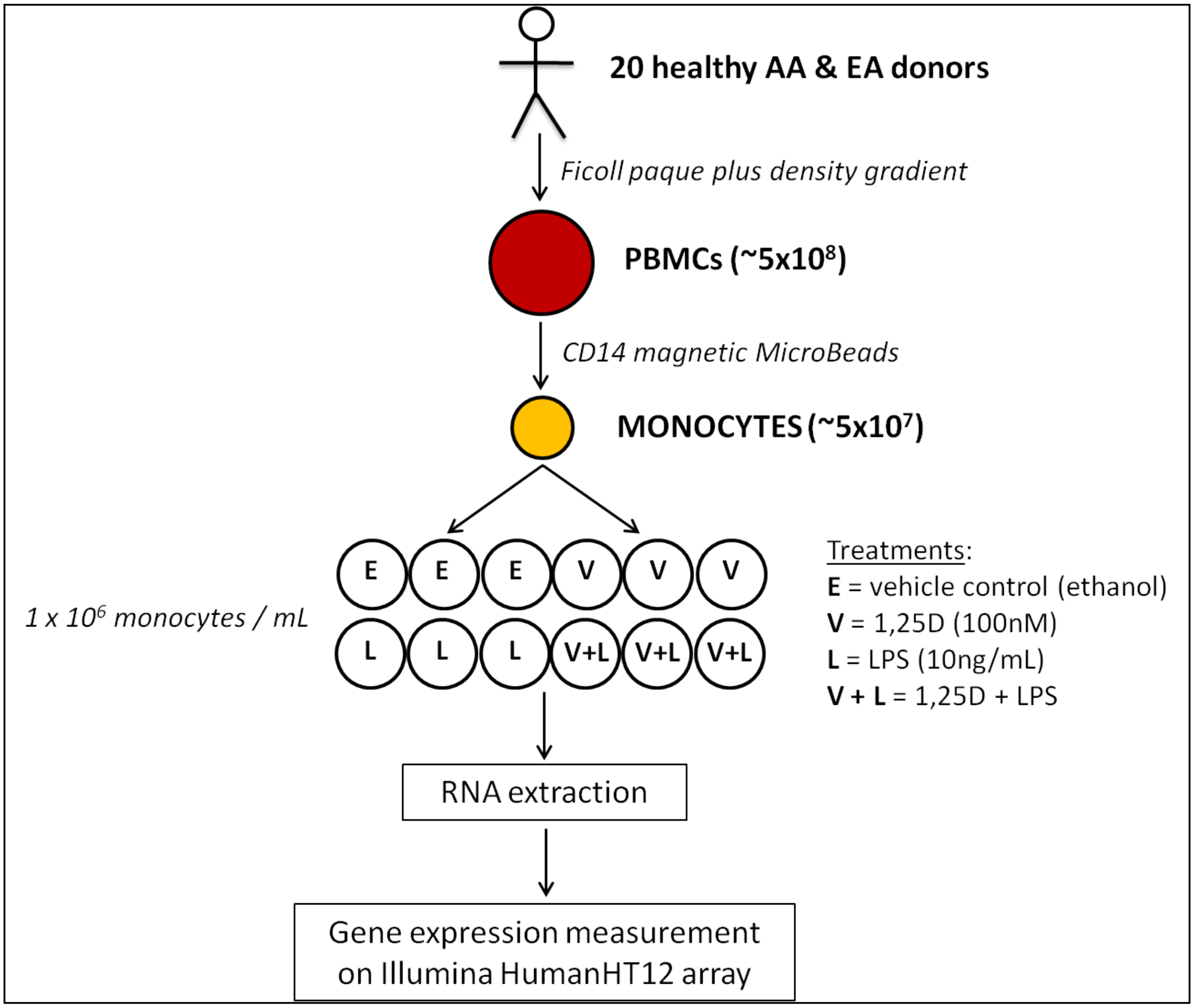

Figure S1: Experimental Design: Primary monocytes were isolated from peripheral blood mononuclear cells (PBMCs) obtained from twenty healthy individuals of African-American (**AA**) and European-American (**EA**) ancestry. The monocytes were cultured in triplicate for 24 hours under four treatment conditions: i. the vehicle control (ethanol or **E**), ii. 1,25D (**V**), iii. LPS (**L**), and iv. 1,25D + LPS (**V + L**). The three replicates for each treatment were pooled for RNA extraction, and genome-wide gene expression was measured using Illumina microarrays.
