# Supplementary material for: Patterns of Transcriptional Response to 1,25-Dihydroxyvitamin D3 and Bacterial Lipopolysaccharide in Primary Human Monocytes
Source: G3 (Bethesda). 2016 Mar 11;6(5):1345–55. doi: 10.1534/g3.116.028712 (PMC4856085; doi:10.1534/g3.116.028712)
Supplement: Supplemental Material [file supp_g3.116.028712_TableS2.pdf]

Table S2: Principal components analysis of variance-stabilized log2-transformed expression data. (**A**) P-values for association between first six principal components (PCs) and sample covariates, with proportion of variance in expression data explained by each principal component at the bottom row. (**B**) R-squared values measuring effect sizes of associations between PCs and covariates.

A.

**P-values**	**PC1**	**PC2**	**PC3**	**PC4**	**PC5**	**PC6**
Sample ID	0.966	0.993	0.205	0.094	5.01 x10^{-5}	6.17 x10^{-5}
Array Batch	0.164	0.265	4.95 x10^{-6}	3.44 x10^{-16}	0.392	0.757
Treatment	6.08 x10^{-17}	4.68 E-06	0.332	0.334	0.695	0.711
Month	0.303	0.801	0.834	0.001	0.043	1.0 x10^{-3}
Age	0.853	0.946	1.36 x10^{-6}	0.617	4.0 x10^{-3}	0.097
Gender	0.314	0.547	0.272	0.863	0.124	0.739
Ancestry	0.711	0.911	0.399	0.403	0.047	0.004
Serum 25D	0.205	0.920	1.0 x10^{-3}	0.147	0.743	0.929
Serum PTH	0.569	0.615	6.1 x10^{-6}	0.055	0.758	0.336
RNA concentration	1.54 x10^{-5}	0.986	0.029	0.158	0.935	0.061
RIN	0.160	9.0 x10^{-3}	5.0 x10^{-3}	0.277	0.524	0.973
	**PC1**	**PC2**	**PC3**	**PC4**	**PC5**	**PC6**
**Proportion of Variance**	0.223	0.086	0.067	0.058	0.039	0.034

B.

**R-squared values**	**PC1**	**PC2**	**PC3**	**PC4**	**PC5**	**PC6**
Sample ID	2.47 x10^{-5}	1.12 x10^{-6}	0.021	0.037	0.196	0.191
Array Batch	0.025	0.016	0.241	0.586	0.010	1.0 x10^{-3}
Treatment	0.604	0.242	0.012	0.012	2.0 x10^{-3}	2.0 x10^{-3}
Month	0.014	1 x10^{-3}	1 x10^{-3}	0.139	0.053	0.140
Age	4.56 x10^{-3}	6.01 x10^{-5}	0.266	3 x10^{-3}	0.103	0.036
Gender	0.013	5.0 x10^{-3}	0.016	3.95 x10^{-4}	0.031	1 x10^{-3}
Ancestry	0.002	1.65 x10^{-4}	9 x10^{-3}	9 x10^{-3}	0.051	0.103
Serum 25D	0.021	1.35 x10^{-4}	0.136	0.027	1.0 x10^{-3}	1.05 x10^{-4}
Serum PTH	0.004	3.0 x10^{-3}	0.237	0.048	1.0 x10^{-3}	0.012
RNA concentration	0.219	3.89 x10^{-6}	0.061	0.026	8.80 x10^{-5}	0.045
RIN	0.026	0.086	0.099	0.016	5 x10^{-3}	1.0 x10^{-4}
