# Supplementary material for: Patterns of Transcriptional Response to 1,25-Dihydroxyvitamin D3 and Bacterial Lipopolysaccharide in Primary Human Monocytes
Source: G3 (Bethesda). 2016 Mar 11;6(5):1345–55. doi: 10.1534/g3.116.028712 (PMC4856085; doi:10.1534/g3.116.028712)
Supplement: Supplemental Material [file supp_g3.116.028712_TableS3.pdf]

Table S3: Principal components analysis of variance-stabilized log2-transformed expression data adjusted for covariates. (**A**) P-values for association between first six principal components (PCs) and sample covariates, with proportion of variance in expression data explained by each principal component at the bottom row. (**B**) R-squared values measuring effect sizes of associations between PCs and covariates.

A.

**P-values**	**PC1**	**PC2**	**PC3**	**PC4**	**PC5**	**PC6**
Sample ID	0.937	0.716	4.5×10^{-7}	7.0×10^{-3}	0.041	3.50×10^{-4}
Array Batch	0.853	0.865	0.961	0.938	0.963	0.989
Treatment	1.62×10^{-17}	2.76×10^{-7}	0.715	0.402	0.865	0.996
Month	0.829	0.843	0.955	0.927	0.956	0.987
Age	0.634	0.714	0.810	0.886	0.882	0.799
Gender	0.969	0.940	0.081	0.151	0.299	0.056
Ancestry	0.866	0.689	1.04×10^{-5}	1.0×10^{-3}	0.017	1.66×10^{-6}
Serum 25D	0.518	0.888	0.037	0.129	0.253	0.026
Serum PTH	0.381	0.992	0.536	0.850	0.851	0.565
RNA concentration	3.56×10^{-5}	0.754	0.667	0.045	0.378	0.820
RIN score	0.345	3.0×10^{-3}	0.516	0.576	0.374	0.499
	**PC1**	**PC2**	**PC3**	**PC4**	**PC5**	**PC6**
**Proportion of Variance**	0.248	0.109	0.050	0.042	0.034	0.031

B.

**R-squared values**	**PC1**	**PC2**	**PC3**	**PC4**	**PC5**	**PC6**
Sample ID	8.34×10^{-5}	2.0×10^{-3}	**0.286**	0.090	0.054	0.156
Array Batch	4.52×10^{-4}	3.83×10^{-4}	3.16×10^{-5}	8.09×10^{-5}	2.92×10^{-5}	2.53×10^{-6}
Treatment	**0.617**	**0.295**	2.0×10^{-3}	9.0×10^{-3}	3.83×10^{-4}	3.50×10^{-7}
Month	0.001	1.0×10^{-3}	4.30×10^{-5}	1.10×10^{-4}	3.97×10^{-5}	3.44×10^{-6}
Age	0.003	2.0×10^{-3}	1.0×10^{-3}	2.72×10^{-4}	2.94×10^{-4}	1.0×10^{-3}
Gender	1.99×10^{-5}	7.58×10^{-5}	0.040	0.027	0.014	0.047
Ancestry	3.79×10^{-4}	2.0×10^{-3}	**0.227**	0.140	0.073	**0.262**
Serum 25D	6.0×10^{-3}	2.62×10^{-4}	0.056	0.030	0.017	0.063
Serum PTH	0.010	1.31×10^{-6}	5.0×10^{-3}	4.76×10^{-4}	4.64×10^{-4}	4.0×10^{-3}
RNA concentration	**0.203**	1.0×10^{-3}	2.0×10^{-3}	0.052	0.010	1.0×10^{-3}
RIN score	0.012	**0.113**	6.0×10^{-3}	4.0×10^{-3}	0.010	6.0×10^{-3}
