# Supplementary material for: Patterns of Transcriptional Response to 1,25-Dihydroxyvitamin D3 and Bacterial Lipopolysaccharide in Primary Human Monocytes
Source: G3 (Bethesda). 2016 Mar 11;6(5):1345–55. doi: 10.1534/g3.116.028712 (PMC4856085; doi:10.1534/g3.116.028712)
Supplement: Supplemental Material [file supp_g3.116.028712_TableS4.pdf]

Table S4: Biological pathways enriched at a FDR < 0.05 among genes significantly DE in response to single treatment with 1,25D or LPS, identified using linear mixed-effects model. The biological pathways are stratified by direction of transcription response, where up-regulated genes are indicated in **green** while down-regulated genes are indicated in **red**. The biological pathways are also grouped according to the similar response patterns to 1,25D in the **V vs. E** (1,25D relative to vehicle) and **V + L vs. L** (1,25D+LPS relative to LPS) treatment categories, and the similar response patterns to LPS in the **L vs. E** (LPS relative to vehicle) and **V + L vs. E** (1,25D+LPS relative to vehicle) treatment categories.

**Biological Pathway**	**V vs. E**	**V + L vs. L**	**L vs. E**	**V + L vs. E**
EIF2 Signaling	green	green	red	red
mTOR Signaling	green	green		
Oxidative Phosphorylation	green		red	red
Mitochondrial Dysfunction	green		red	red
Adipogenesis pathway		green		green
Insulin Receptor Signaling		green		green
fMLP Signaling in Neutrophils	red	red	green	
NRF2-mediated Oxidative Stress Response	red	red	green	
Signaling by Rho Family GTPases	red	red		
Role of NFAT in Regulation of the Immune Response	red	red	green	green
Chemokine Signaling	red	red		
Remodeling of Epithelial Adherens Junctions	red	red	green	
Antigen Presentation Pathway	red	red		
Androgen Signaling	red	red	green	green
Germ Cell-Sertoli Cell Junction Signaling	red	red	green	
Phagosome maturation	red	red		
Tec Kinase Signaling	red		green	
Phospholipase C Signaling	red		green	
Integrin Signaling	red		green	green
Role of JAK1, JAK2 and TYK2 in Interferon Signaling	red		green	
T Helper Cell Differentiation	red		green	green
Production of Nitric Oxide and Reactive Oxygen Species in Macrophages	red		green	green
OX40 Signaling Pathway	red			green
PI3K Signaling in B Lymphocytes	red		green	green
IL-8 Signaling	red		green	green
Role of Pattern Recognition Receptors in Recognition of Bacteria and Viruses	red		green	green
Dendritic Cell Maturation	red		green	green
CD28 Signaling in T Helper Cells	red			green
B Cell Receptor Signaling	red		green	green
Communication between Innate and Adaptive Immune Cells	red		green	green
NF-kB Signaling	red		green	green
Mismatch Repair in Eukaryotes	red			red

G-Protein Coupled Receptor Signaling				
Protein Ubiquitination Pathway				
IL-4 Signaling				
Leukocyte Extravasation Signaling				
IL-10 Signaling				
IL-6 Signaling				
Acute Phase Response Signaling				
TNFR2 Signaling				
TNFR1 Signaling				
Glucocorticoid Receptor Signaling				
CD40 Signaling				
IL-17 Signaling				
LPS-stimulated MAPK Signaling				
Toll-like Receptor Signaling				
JAK/Stat Signaling				
iNOS Signaling				
B Cell Activating Factor Signaling				
Apoptosis Signaling				
IL-15 Signaling				
CD27 Signaling in Lymphocytes				
IL-1 Signaling				
IL-2 Signaling				
Role of JAK family kinases in IL-6-type Cytokine Signaling				
T Cell Receptor Signaling				
IL-9 Signaling				
Oncostatin M Signaling				
IL-22 Signaling				
IL-12 Signaling and Production in Macrophages				
VDR/RXR Activation				
Differential Regulation of Cytokine Production in Macrophages and T Helper Cells by IL-17A and IL-17F				
tRNA Charging				
Fatty Acid beta-oxidation I				
Galactose Degradation I (Leloir Pathway)				
