# Supplementary material for: Patterns of Transcriptional Response to 1,25-Dihydroxyvitamin D3 and Bacterial Lipopolysaccharide in Primary Human Monocytes
Source: G3 (Bethesda). 2016 Mar 11;6(5):1345–55. doi: 10.1534/g3.116.028712 (PMC4856085; doi:10.1534/g3.116.028712)
Supplement: Supplemental Material [file supp_g3.116.028712_TableS5.pdf]

Table S5: Biological pathways enriched at a FDR < 0.05 among genes in the different Cormotif patterns. The pathways are stratified by direction of transcription response in each Cormotif pattern, where up-regulated genes are indicated in **green** while down-regulated genes are indicated in **red**. For the pathways enriched among genes in the "All" and "All except V+L" patterns, **green** = genes up-regulated by 1,25D and down-regulated by LPS, **red** = genes down-regulated by 1,25D and up-regulated by LPS.

**Biological Pathway**	**All**	**All except V+L**	**1,25D**	**1,25D-all**	**LPS**
EIF2 Signaling	green	green			
Regulation of eIF4 and p70S6K Signaling	green	green			
mTOR Signaling	green	green			
Purine Nucleotides De Novo Biosynthesis II	green				
tRNA Charging	green				red
Spermidine Biosynthesis I	green				
Adenine and Adenosine Salvage I	green				
Inosine-5'-phosphate Biosynthesis II	green				
Oxidative Phosphorylation		green	green		
Mitochondrial Dysfunction		green	green		red
Granulocyte Adhesion and Diapedesis		red			green
IL-8 Signaling		red			green
NF-κB Signaling		red			green
IL-17A Signaling in Fibroblasts		red			green
PPAR Signaling		red			
TNFR2 Signaling		red			green
Role of NFAT in Regulation of the Immune Response		red			green
Unfolded protein response		red			
Mechanisms of Viral Exit from Host Cells		red		red	
4-1BB Signaling in T Lymphocytes		red			green
phagosome maturation		red			
Induction of Apoptosis by HIV1		red			
STAT3 Pathway		red			
TWEAK Signaling		red			
UDP-N-acetyl-D-glucosamine Biosynthesis II		red			
Role of Macrophages, Fibroblasts and Endothelial Cells in Rheumatoid Arthritis		red			green
CXCR4 Signaling		red			
IL-10 Signaling		red			green
Role of Hypercytokinemia/hyperchemokinemia in the Pathogenesis of Influenza		red			
Apoptosis Signaling		red			
Macropinocytosis Signaling			green		
Adipogenesis			green		
Role of JAK family kinases in IL-6-type Cytokine Signaling				green	green

RhoGDI Signaling					
B Cell Receptor Signaling					
Chemokine Signaling					
fMLP Signaling in Neutrophils					
Actin Nucleation by ARP-WASP Complex					
RhoA Signaling					
Integrin Signaling					
Tec Kinase Signaling					
Hereditary Breast Cancer Signaling					
Regulation of Actin-based Motility by Rho					
p70S6K Signaling					
Ephrin Receptor Signaling					
Role of BRCA1 in DNA Damage Response					
NRF2-mediated Oxidative Stress Response					
Signaling by Rho Family GTPases					
Actin Cytoskeleton Signaling					
CD28 Signaling in T Helper Cells					
Axonal Guidance Signaling					
FAK Signaling					
Non-Small Cell Lung Cancer Signaling					
N-acetylglucosamine Degradation II					
CCR3 Signaling in Eosinophils					
Remodeling of Epithelial Adherens Junctions					
Leukocyte Extravasation Signaling					
Fcγ Receptor-mediated Phagocytosis in Macrophages and Monocytes					
Epithelial Adherens Junction Signaling					
IL-4 Signaling					
Glioma Signaling					
IL-6 Signaling					
Role of IL-17A in Arthritis					
IL-17A Signaling in Airway Cells					
Role of IL-17F in Allergic Inflammatory Airway Diseases					
TREM1 Signaling					
CD40 Signaling					
Dendritic Cell Maturation					
IL-17 Signaling					
Acute Phase Response Signaling					
Type II Diabetes Mellitus Signaling					
Glucocorticoid Receptor Signaling					
Role of IL-17A in Psoriasis					
RANK Signaling in Osteoclasts					
Regulation of IL-2 Expression in Activated and Anergic T Lymphocytes					
Agranulocyte Adhesion and Diapedesis					
Type I Diabetes Mellitus Signaling					
Amyloid Processing					
HGF Signaling					
T Helper Cell Differentiation					
NF-κB Activation by Viruses					
Toll-like Receptor Signaling					
IL-22 Signaling					
Hypoxia Signaling in the Cardiovascular System					

Cholecystokinin/Gastrin-mediated Signaling					
EGF Signaling					
Erythropoietin Signaling					
TNFR1 Signaling					
Gαq Signaling					
Role of Tissue Factor in Cancer					
Chondroitin and Dermatan Biosynthesis					
April Mediated Signaling					
PEDF Signaling					
PKCθ Signaling in T Lymphocytes					
Activation of IRF by Cytosolic Pattern Recognition Receptors					
Graft-versus-Host Disease Signaling					
JAK/Stat Signaling					
PI3K Signaling in B Lymphocytes					
LPS-stimulated MAPK Signaling					
B Cell Activating Factor Signaling					
GM-CSF Signaling					
Molecular Mechanisms of Cancer					
Pyridoxal 5'-phosphate Salvage Pathway					
G-Protein Coupled Receptor Signaling					
IL-2 Signaling					
MIF-mediated Glucocorticoid Regulation					
Gα12/13 Signaling					
Acute Myeloid Leukemia Signaling					
Role of Osteoblasts, Osteoclasts and Chondrocytes in Rheumatoid Arthritis					
IL-15 Signaling					
Circadian Rhythm Signaling					
HMGB1 Signaling					
IL-1 Signaling					
Death Receptor Signaling					
RAR Activation					
IL-12 Signaling and Production in Macrophages					
Role of JAK1, JAK2 and TYK2 in Interferon Signaling					
IL-9 Signaling					
Antioxidant Action of Vitamin C					
Wnt/β-catenin Signaling					
Glutaryl-CoA Degradation					
Isoleucine Degradation I					
Tryptophan Degradation III (Eukaryotic)					
Fatty Acid β-oxidation I					
Galactose Degradation I (Leloir Pathway)					
Superpathway of Geranylgeranyldiphosphate Biosynthesis I (via Mevalonate)					
Ketolysis					
Mevalonate Pathway I					
Heme Biosynthesis II					
Cell Cycle Control of Chromosomal Replication					
Folate Transformations I					
TCA Cycle II (Eukaryotic)					
Superpathway of Methionine Degradation					
Tetrapyrrole Biosynthesis II					

Folate Polyglutamylation					
Ketogenesis					
Dolichyl-diphosphooligosaccharide Biosynthesis					
