# Supplementary material for: Patterns of Transcriptional Response to 1,25-Dihydroxyvitamin D3 and Bacterial Lipopolysaccharide in Primary Human Monocytes
Source: G3 (Bethesda). 2016 Mar 11;6(5):1345–55. doi: 10.1534/g3.116.028712 (PMC4856085; doi:10.1534/g3.116.028712)
Supplement: Supplemental Material [file supp_g3.116.028712_TableS6.pdf]

Table S6: Diseases enriched among down-regulated genes in the "1,25D-all" Cormotif pattern at a FDR < 0.05. **B-H p-value*** = Benjamini-Hochberg multiple testing corrected p-value.

**Disease Category**	**B-H p-value***	**Genes down-regulated in "1,25D-all" Cormotif pattern**
Inflammatory Response	6.24 x 10^{-5}	*DPYSL2,GAS6,SGK1,DUSP3,TMSB10/TMSB4X,ICOSLG/LOC102723996,CFL1,RRAS,PLEC,ANXA2,RAP1A,ABCA7,OGG1,MTMR4,CAPN1,ALCAM,PIK3CD,ARHGAP1,ACTG1,CNN2,HAMP,RAB32,POU2F2,PRDX1,IL21R,HYOU1,TNFSF10,MMP25,ANPEP,DUSP2,SASH3,TNFSF12,DOCK2,ORAI1,RNASE2,C12orf4,IFNGR2,GNAQ,TOB1,CD58,NRROS,KIAA0226,GPR183,ABHD6,GNAI3,LY96,WAS,IMPDH1,TNFSF13,RBPJ,ARHGDIA,CD81,CH25H,RGS1,HYAL2,NAGK,TNFRSF4,PFN1,IDI1,IL12RB1,HSPA5,CD300A,RHOB,LTBR,SH3KBP1,STK17B,S100A10,NBEAL2,P4HB,IL2RG,PTPN6,BMP2K,MALT1,NFKBID,FZD5,MGAT2,ABCD1,SPRED1,CCL24,OTULIN,HSP90B1,CCDC88A,AHNAK,DUSP10,CD22,PRKCA,TPMT,CALR,NAIP,IRF4,PRMT2,AP3D1,SMAD7,SOD1,PLXND1,TNFRSF14,BTK,ZBTB46,CORO1A,TLR6,CTSC,DNM1L,LGALS1,MSN*
Immunological Disease	1.55 x 10^{-3}	*RFXANK,CD81,DPYSL2,RGS1,NAGK,TNFRSF4,IDI1,IL12RB1,GAS6,SGK1,PTTG1,DDB2,HSPA5,RB1,SEC24D,RHOB,OLIG2,XPO1,LTBR,ICOSLG/LOC102723996,STK17B,NBEAL2,P4HB,IL2RG,PTPN6,CFL1,RRAS,DLEU2,TSPAN33,MALT1,ARHGDIB,ABCA7,OGG1,DVL2,MTMR4,CAPN1,CECR6,BTG2,RASSF4,ALCAM,PIK3CD,ARHGAP1,ACTG1,POU2F2,PRDX1,IL21R,DAGLA,TNFSF10,DUSP2,TTC37,AHNAK,TNFSF12,DUSP10,DOCK2,EAF2,CD22,PALD1,BLM,ORAI1,PRKCA,CALR,ATP1B1,IRF4,TGFBI,SMAD7,IFNGR2,TOB1,CD58,SOD1,TNFRSF14,BTK,ZBTB46,CRELD2,SIRT2,MUM1,WAS,TNFSF13,TLR6,CORO1A,ARHGDIA,RBPJ,FEN1,DNM1L,SEPT6,MSN,LGALS1*
Inflammatory Disease	1.55 x 10^{-3}	*TNFRSF4,GAS6,IL21R,TNFSF10,HSPA5,HSP90B1,DUSP10,LTBR,STK17B,PRKCA,CALR,NBEAL2,IL2RG,PTPN6,IRF4,RRAS,SMAD7,TOB1,TSPAN33,SOD1,ABCA7,TNFRSF14,BTK,CORO1A,ALCAM,RBPJ,LGALS1*
Neurological Disease	1.55 x 10^{-3}	*SPRED1,PFN1,TNFRSF4,VPS35,GAS6,IL21R,CWF19L1,TNFSF10,SETX,HSPA5,HSP90B1,FANCD2,DUSP10,DHTKD1,FGD4,LTBR,RTN2,STK17B,PRKCA,CALR,IL2RG,IRF4,TGFBI,RRAS,GNAQ,TOB1,LRSAM1,LZTR1,SOD1,TNFRSF14,KIAA0226,SLC33A1,CORO1A,ALCAM,RBPJ,LGALS1*
Hematological Disease	4.78 x 10^{-3}	*HYAL2,TNFRSF4,SGK1,GAS6,PTTG1,DDB2,HSPA5,RB1,SEC24D,RHOB,OLIG2,XPO1,LTBR,STK17B,NBEAL2,IL2RG,CFL1,DLEU2,TSPAN33,ANXA2,MALT1,ABCA7,OGG1,DVL2,MTMR4,CECR6,CAPN1,BTG2,RASSF4,PIK3CD,ACTG1,ARHGAP1,POU2F2,PRDX1,IL21R,TNFSF10,DAGLA,MKL1,HSP90B1,DUSP10,EAF2,DOCK2,CD22,PALD1,BLM,TPMT,IRF4,TGFBI,IFNGR2,CD58,SOD1,TNFRSF14,BTK,CRELD2,SIRT2,WAS,MUM1,TLR6,FEN1,DNM1L,SEPT6,LGALS1*
Hereditary Disorder	2.32 x 10^{-2}	*RFXANK,PFN1,VPS35,PTTG1,CWF19L1,SETX,HSPA5,HSP90B1,AHNAK,TNFSF12,DOCK2,DHTKD1,FGD4,RTN2,ORAI1,CALR,IL2RG,PTPN6,TGFBI,PLEC,LRSAM1,SOD1,ARHGDIB,KIAA0226,BTK,SLC33A1,IMPDH1,PIK3CD,ARHGDIA,ARHGAP1*
Hypersensitivity Response	3.52 x 10^{-2}	*BTK,IL2RG,TNFRSF4,MTMR4,WAS,CAPN1,CORO1A,C12orf4,PIK3CD,DUSP2,CD300A,ORAI1*
Cancer	3.52 x 10^{-2}	*CD81,TUBA1B,PHLDA1,ARHGAP26,TNFRSF4,GAS6,NDRG2,SGK1,PTTG1,KLF6,MCUR1,DDB2,POTEG (includes others),HSPA5,CACYBP,VASH1,RB1,SEC24D,RHOB,OLIG2,DHTKD1,XPO1,LTBR,PLCL1,TMSB10/TMSB4X,ICOSLG/LOC102723996,STK17B,S100A10,NBEAL2,PTPN6,IL2RG,CFL1,DLEU2,PLEC,TSPAN33,SIPA1L2,ANXA2,ARHGDIB,ABCA7,OGG1,DVL2,MTMR4,TES,CECR6,BTG2,RASSF4,KIDINS220,ALCAM,KLHL12,PIK3CD,ACTG1,EMILIN2,POU2F2,PRDX1,IL21R,HMGN1,HYOU1,DAGLA,TN*

		FSF10,MKL1,ANPEP,USO1,CCDC88A,HSP90B1,ACTR3,FANCD2,TNFSF12,DOCK2,EAF2,CD22,PALD1,BLM,PRKCA,ORAI1,CALR,IRF4,MAP3K6,TGFBI,DROSHA,SMAD7,CD58,SOD1,TNFRSF14,BTK,LACC1,ZBTB46,CRELD2,SIRT2,WAS,MUM1,IMPDH1,TNFSF13,TLR6,FEN1,DNM1L,CTSC,SEPT6,LGALS1
Organismal Injury and Abnormalities	3.52×10^{-2}	*TUBA1B,GAS6,SGK1,DDB2,CACYBP,RB1,SEC24D,OLIG2,TMSB10/TMSB4X,PLCL1,ICOSLG/LOC102723996,CFL1,PLEC,ANXA2,SIPA1L2,TSPAN33,ABCA7,OGG1,ARHGDIB,DVL2,MTMR4,BTG2,KIDINS220,ALCAM,PIK3CD,ARHGAP1,ACTG1,EMILIN2,PRDX1,POU2F2,IL21R,HMGN1,HYOU1,TNFSF10,DAGLA,ANPEP,SASH3,USO1,ACTR3,TNFSF12,DOCK2,PALD1,ORAI1,TGFBI,DROSHA,GNAQ,TOB1,CD58,ZG16B,MUM1,WAS,TNFSF13,IMPDH1,ARHGDIA,FEN1,RBPJ,CD81,PHLDA1,TNFRSF4,ARHGAP26,NDRG2,PTTG1,KLF6,MCUR1,POTEG (includes others),HSPA5,VASH1,RHOB,DHTKD1,XPO1,LTBR,STK17B,S100A10,NBEAL2,PTPN6,IL2RG,DLEU2,LZTR1,TES,CECR6,RASSF4,KLHL12,SPRED1,MKL1,CCDC88A,HSP90B1,FANCD2,EAF2,CD22,BLM,PRKCA,CALR,IRF4,MAP3K6,SMAD7,SOD1,TNFRSF14,BTK,LACC1,CRELD2,ZBTB46,SIRT2,TLR6,ARHGAP31,DNM1L,CTSC,SEPT6,LGALS1*
Skeletal and Muscular Disorders	4.43×10^{-2}	*CD81,CALR,RB1,HSP90B1,AHNAK,RRAS,PLEC,HSPA5,NDN*
Developmental Disorder	4.64×10^{-2}	*RFXANK,CALR,IL2RG,SPRED1,TGFBI,PTTG1,GNAQ,LZTR1,HSPA5,ARHGDIB,SASH3,BTK,HSP90B1,WAS,DOCK2,ARHGAP31,RBPJ,PIK3CD,ARHGDIA,ARHGAP1,ORAI1,PRKCA*
