# Supplementary material for: Patterns of Transcriptional Response to 1,25-Dihydroxyvitamin D3 and Bacterial Lipopolysaccharide in Primary Human Monocytes
Source: G3 (Bethesda). 2016 Mar 11;6(5):1345–55. doi: 10.1534/g3.116.028712 (PMC4856085; doi:10.1534/g3.116.028712)
Supplement: Supplemental Material [file supp_g3.116.028712_TableS7.pdf]

**Table S7:** Enrichment of VDR ChIP-seq peaks among genes responsive to 1,25D treatment. VDR ChIP-seq peaks were obtained from published datasets, while 1,25D responsive genes were obtained among those that were significantly differentially expressed in response to different 1,25D treatment conditions, from the linear mixed-effects and Cormotif analyses. Enrichment of VDR peaks was calculated using Fisher's exact test, comparing DE genes to non-DE genes.

| **Treatment** | **DE genes** | **DE genes with VDR binding site** | **Proportion of DE genes with VDR binding site** | **Non-DE genes** | **Non-DE genes with VDR binding site** | **Proportion of Non-DE genes with VDR binding site** | **Enrichment p-values** |
|---|---|---|---|---|---|---|---|
| **Linear mixed-effects model** | | | | | | | |
| V vs. E | 2887 | 202 | 0.07 | 8071 | 300 | 0.04 | $4.56 \times 10^{-11}$ |
| V + L vs. E | 4720 | 335 | 0.07 | 6238 | 280 | 0.04 | $3.97 \times 10^{-8}$ |
| V + L vs. L | 2405 | 209 | 0.09 | 8554 | 466 | 0.05 | $1.54 \times 10^{-7}$ |
| **Cormotif Analysis** | | | | | | | |
| 1,25D response ("All", "All except V+L", "1,25D" and 1,25D-all" Cormotifs) | 2761 | 189 | 0.07 | 5737 | 186 | 0.03 | $3.28 \times 10^{-12}$ |
| 1,25D response: ("1,25D" and 1,25D-all" Cormotifs) | 1132 | 114 | 0.10 | 5737 | 186 | 0.03 | $3.33 \times 10^{-18}$ |
