# Supplementary material for: Patterns of Transcriptional Response to 1,25-Dihydroxyvitamin D3 and Bacterial Lipopolysaccharide in Primary Human Monocytes
Source: G3 (Bethesda). 2016 Mar 11;6(5):1345–55. doi: 10.1534/g3.116.028712 (PMC4856085; doi:10.1534/g3.116.028712)
Supplement: Supplemental Material [file supp_g3.116.028712_FigureS2.pdf]

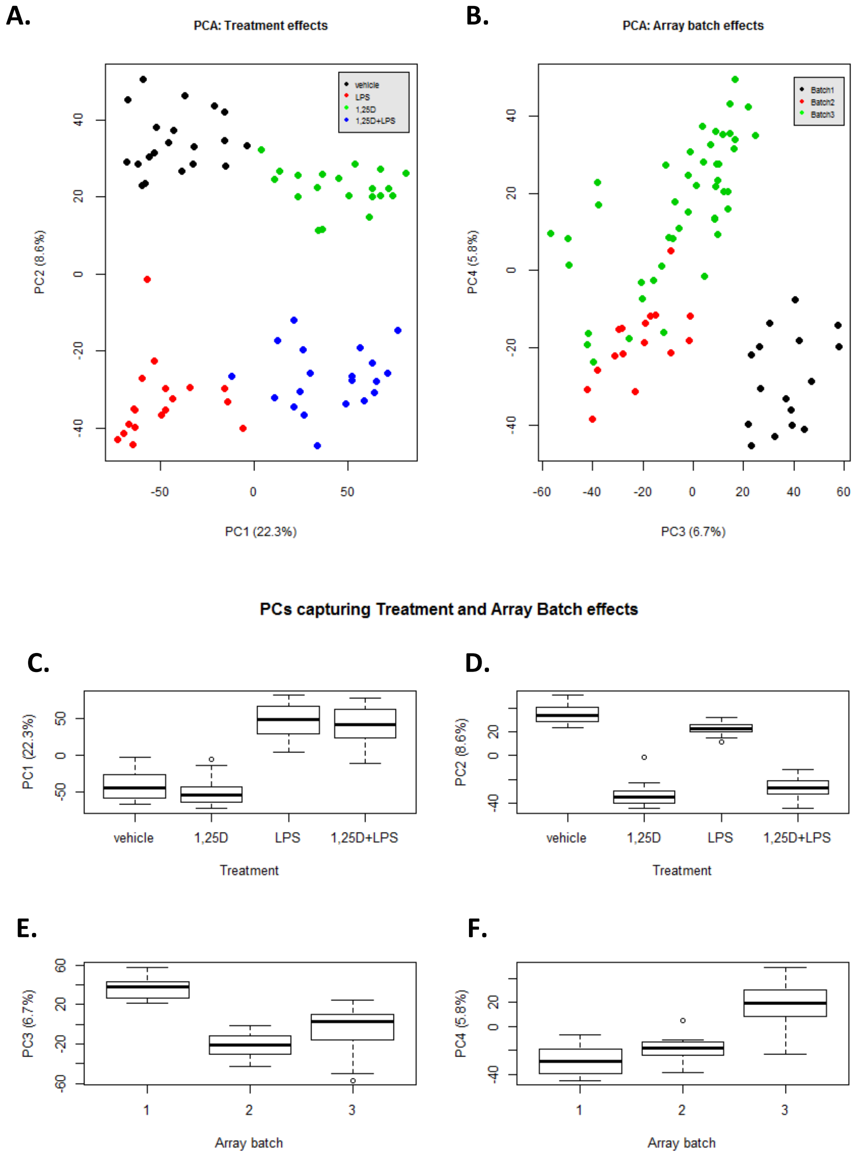

Figure S2: Principal components analysis (PCA) of the expression data indicating the sources of transcriptome-wide variation. (**A**) Plot of PC1 vs. PC2, both of which captured the effects of treatment on the transcriptome. (**B**) Plot of PC3 vs. PC4, both of which captured array batch effects. (**C**) Boxplot showing effects of LPS treatment on expression data captured by PC1. (**D**) Boxplot showing effect of 1,25D treatment on expression data captured by PC2. (**E**) The array batch effects were captured by PC3 (**E**), and PC4 (**F**). The proportion of variation explained by the PCs is in parentheses.
