# Supplementary material for: Patterns of Transcriptional Response to 1,25-Dihydroxyvitamin D3 and Bacterial Lipopolysaccharide in Primary Human Monocytes
Source: G3 (Bethesda). 2016 Mar 11;6(5):1345–55. doi: 10.1534/g3.116.028712 (PMC4856085; doi:10.1534/g3.116.028712)
Supplement: Supplemental Material [file supp_g3.116.028712_FigureS4.pdf]

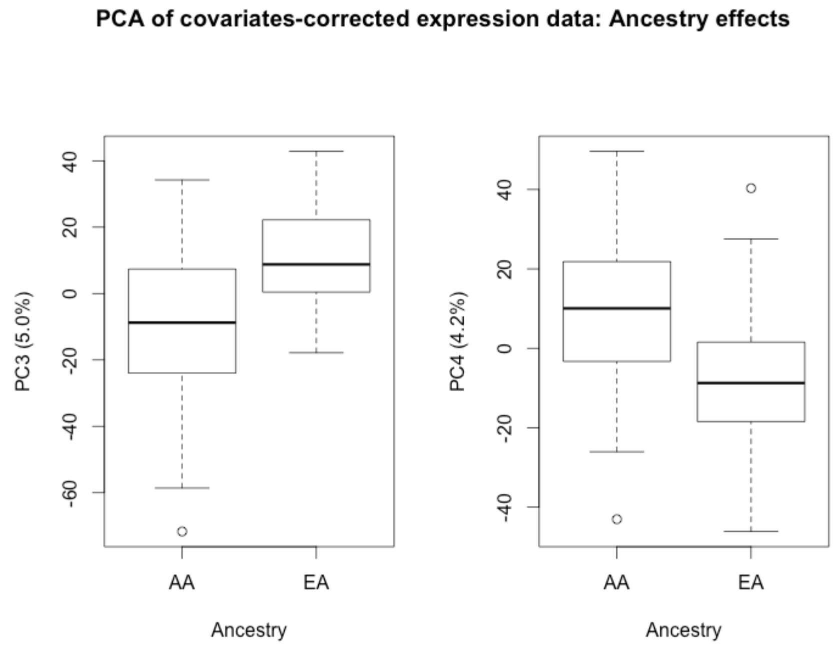

Figure S4: Boxplot showing inter-ethnic variation in covariates-corrected expression data captured by PC3 and PC4, with the proportion of variation explained in parenthesis.
