# Supplementary material for: Patterns of Transcriptional Response to 1,25-Dihydroxyvitamin D3 and Bacterial Lipopolysaccharide in Primary Human Monocytes
Source: G3 (Bethesda). 2016 Mar 11;6(5):1345–55. doi: 10.1534/g3.116.028712 (PMC4856085; doi:10.1534/g3.116.028712)
Supplement: Supplemental Material [file supp_g3.116.028712_FigureS5.pdf]

A.

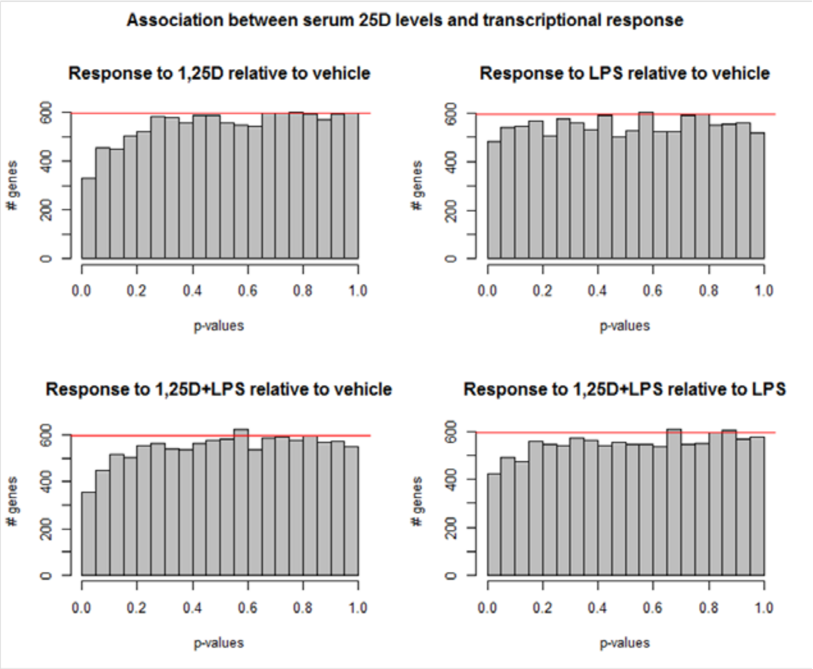

B.

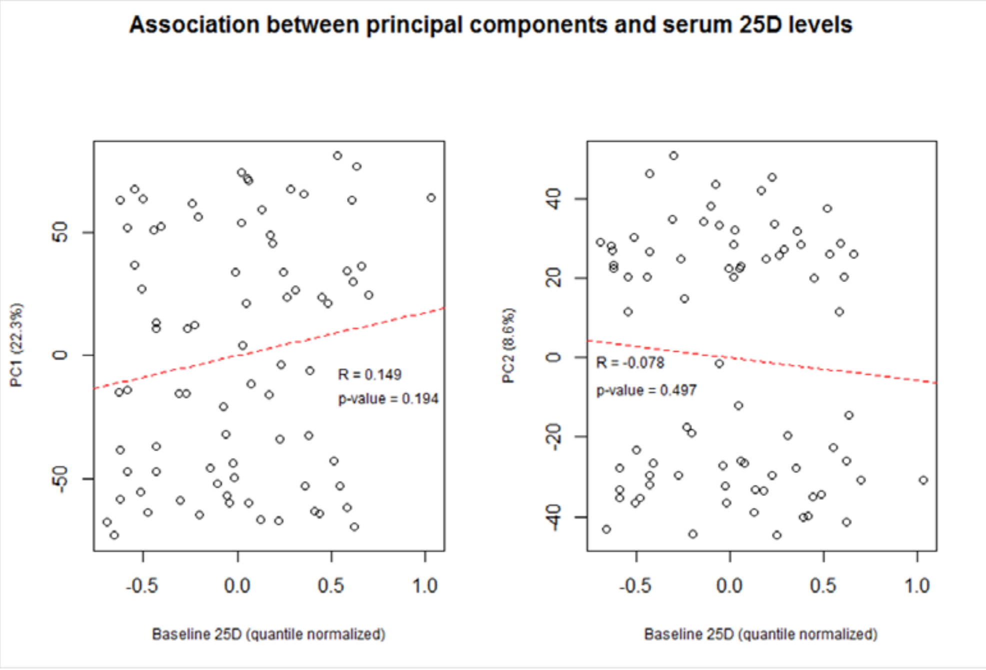

Figure S5: Examining the effect of baseline 25D levels on transcriptional response. (**A**) Distribution of p-values from simple linear model measuring association between baseline 25D levels and log-fold change response to each of the four treatment conditions (1,25D, LPS, 1,25D+LPS relative to vehicle, and 1,25D+LPS relative to LPS). (**B**) Correlation between principal components 1 and 2 (PC1 and PC2), and baseline 25D levels. PC1 and PC2 captured the effect of LPS and 1,25D on the transcriptome.
