# Supplementary material for: Patterns of Transcriptional Response to 1,25-Dihydroxyvitamin D3 and Bacterial Lipopolysaccharide in Primary Human Monocytes
Source: G3 (Bethesda). 2016 Mar 11;6(5):1345–55. doi: 10.1534/g3.116.028712 (PMC4856085; doi:10.1534/g3.116.028712)
Supplement: Supplemental Material [file supp_g3.116.028712_FigureS6.pdf]

## A: Complex I

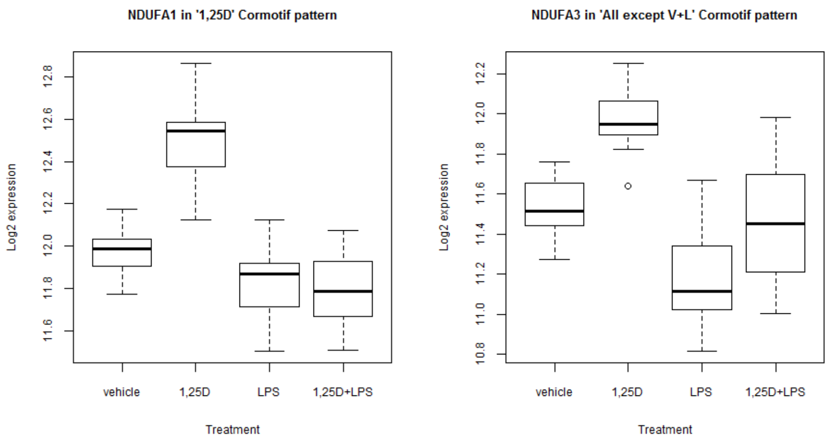


## B: Complex II

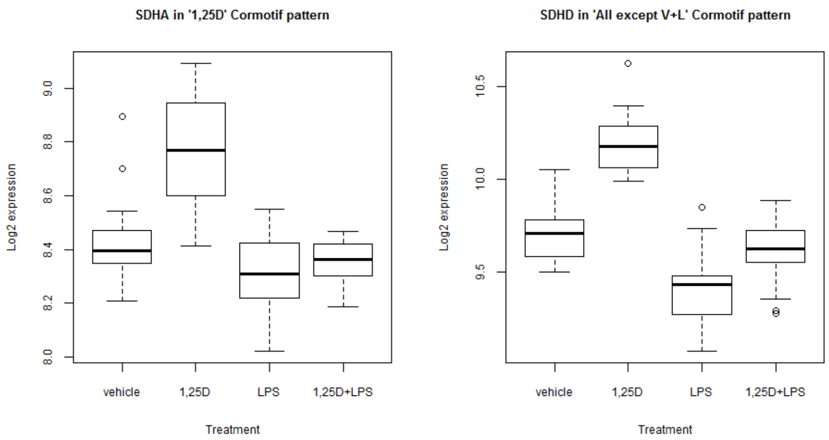


## C: Complex III

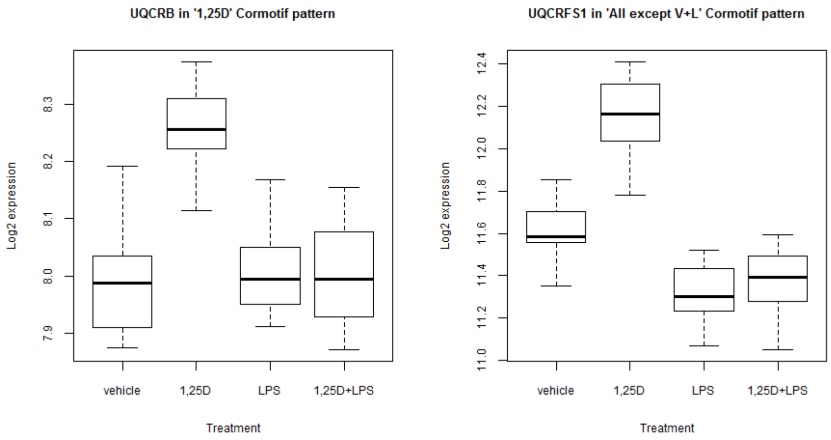

**D: Complex IV**

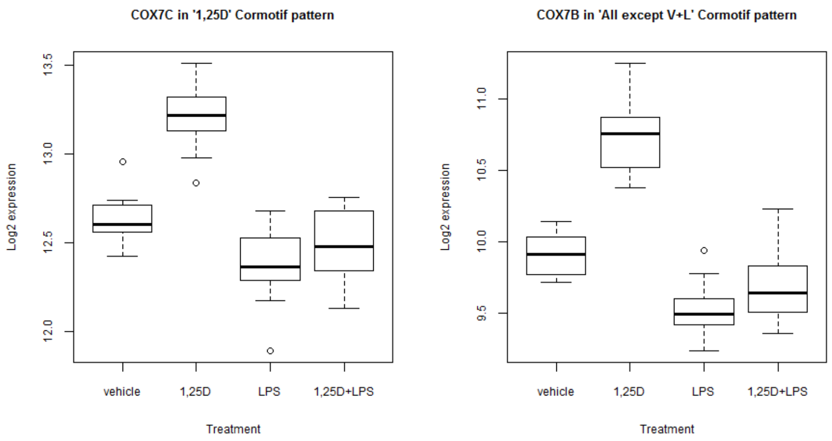


**E: Complex V**

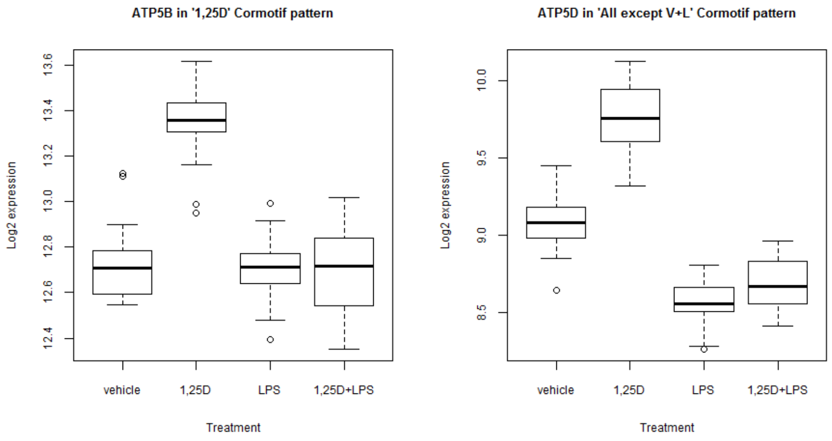


**Figure S6**: Boxplots of genes in the Oxidative Phosphorylation pathway clustered in the “1,25D” and “All except V+L” Cormotif patterns with treatment-specific response patterns. Genes representing the five respiratory complexes are shown in **A-E**. Oxidative phosphorylation pathway genes in the “1,25D” pattern were responsive only to 1,25D, while genes in the “All except V+L” pattern were responsive to both 1,25D and LPS in opposite directions.
