# Supplementary material for: Patterns of Transcriptional Response to 1,25-Dihydroxyvitamin D3 and Bacterial Lipopolysaccharide in Primary Human Monocytes
Source: G3 (Bethesda). 2016 Mar 11;6(5):1345–55. doi: 10.1534/g3.116.028712 (PMC4856085; doi:10.1534/g3.116.028712)
Supplement: Supplemental Material [file supp_g3.116.028712_FigureS7.pdf]

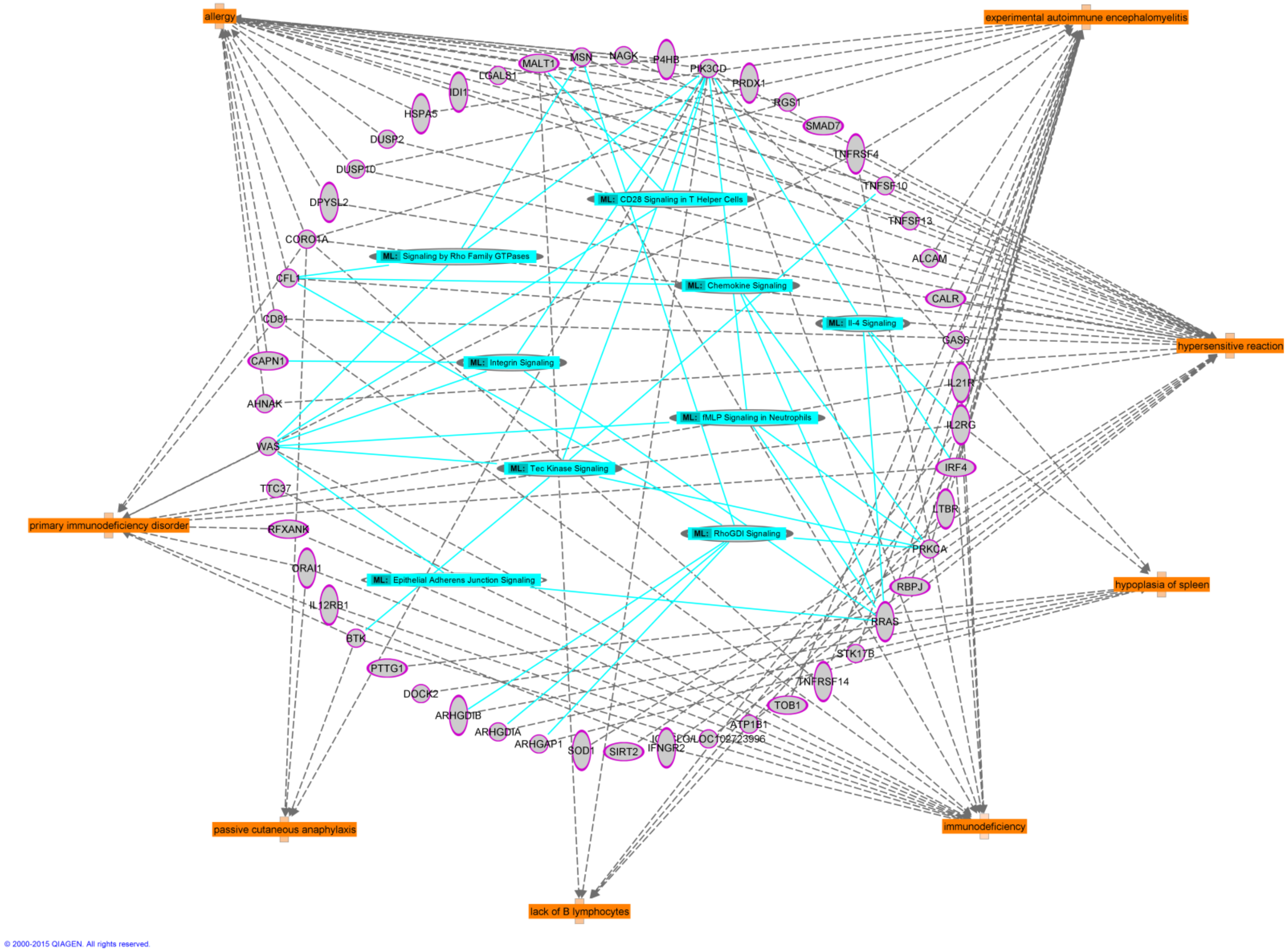

Figure S7: Network of down-regulated genes in the “1,25D-all” Cormotif pattern. Biological pathways enriched among these genes are highlighted in cyan. Immunological and Inflammatory diseases enriched among these genes are highlighted in orange, while genes associated with diseases are circled in purple.
