# Supplementary material for: Patterns of Transcriptional Response to 1,25-Dihydroxyvitamin D3 and Bacterial Lipopolysaccharide in Primary Human Monocytes
Source: G3 (Bethesda). 2016 Mar 11;6(5):1345–55. doi: 10.1534/g3.116.028712 (PMC4856085; doi:10.1534/g3.116.028712)
Supplement: Supplemental Material [file supp_g3.116.028712_FigureS8.pdf]

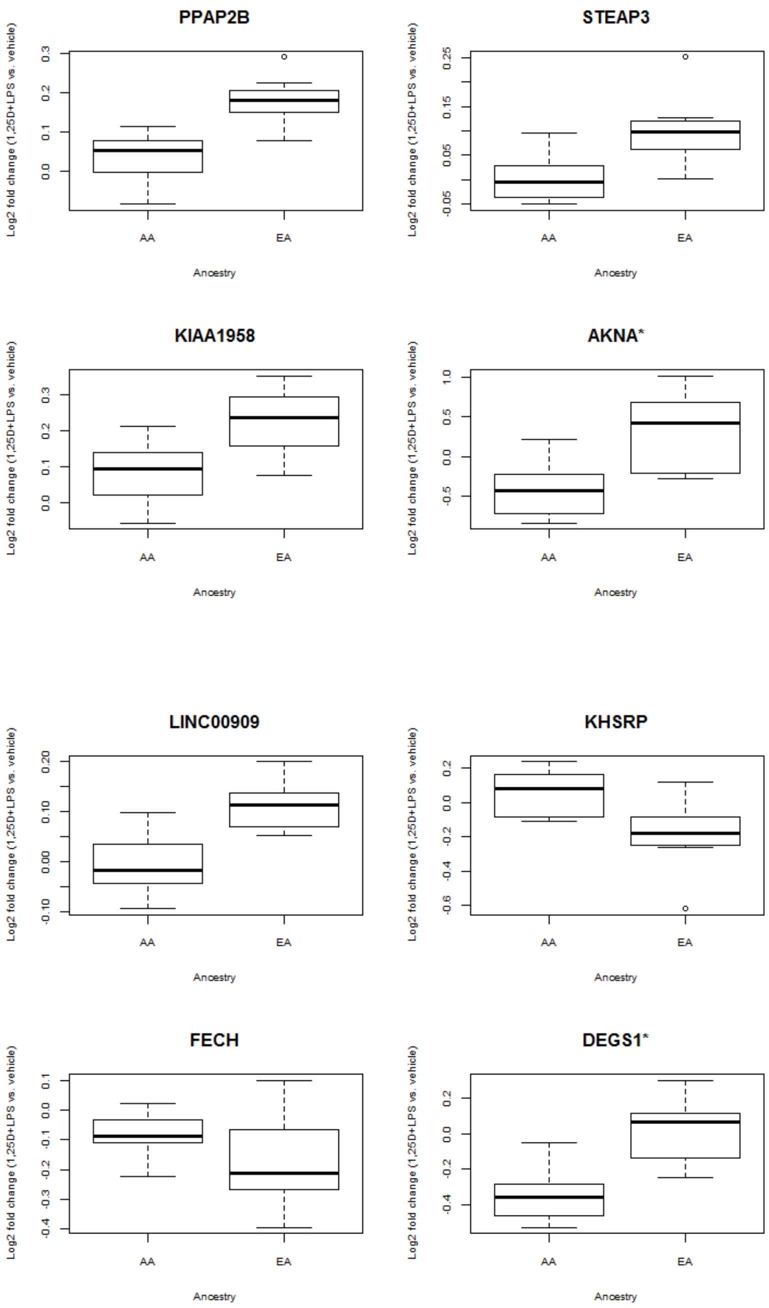
PPAP2B
STEAP3
KIAA1958
AKNA*
LINC00909
KHSRP
FECH
DEGS1*
Log2 fold change (1,25D+LPS vs. vehicle)
AA
EA
Ancestry

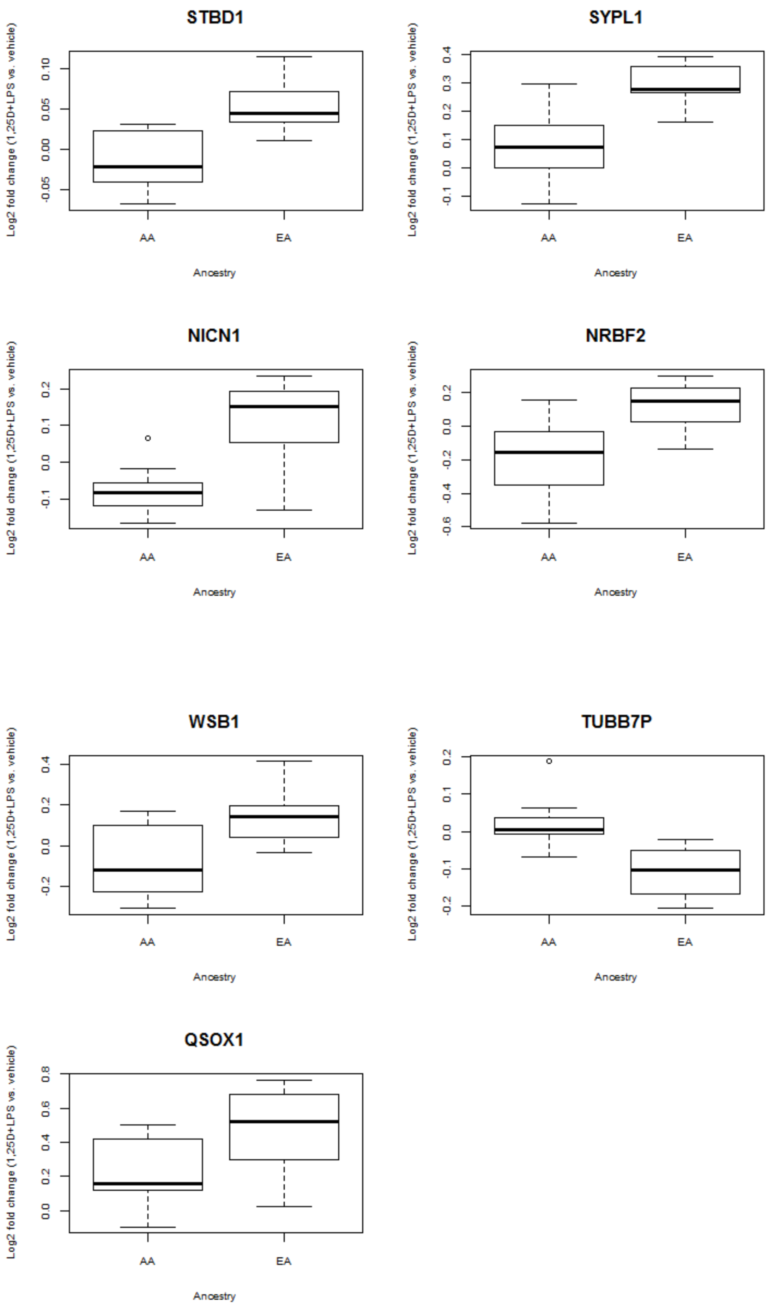

Figure S8: Boxplots of genes with different log fold change in transcript levels between the two ethnic groups in response to 1,25D+LPS relative to vehicle (V + L vs. E) at a FDR < 0.10. 13 genes showed stronger response in EA's, while the 2 genes indicated with asterisks (*AKNA*, and *DEGS1*) showed stronger response in AA's. **AA** = African-American; **EA** = European-American.
