# Supplementary material for: Patterns of Transcriptional Response to 1,25-Dihydroxyvitamin D3 and Bacterial Lipopolysaccharide in Primary Human Monocytes
Source: G3 (Bethesda). 2016 Mar 11;6(5):1345–55. doi: 10.1534/g3.116.028712 (PMC4856085; doi:10.1534/g3.116.028712)
Supplement: Supplemental Material [file supp_g3.116.028712_TableS1.pdf]

Table S1: Comparing averaged sample covariates data across ancestries, with p-values obtained from t-test.

**Covariates**	**EA**	**AA**	**p-value**
Age	29.7	30.1	0.90
Gender (F/M)	3/7	0/10	2.04×10^{-4}
Serum 25D concentration (nM)	52.8	35.3	6.92×10^{-6}
Serum PTH (pM)	27.6	32.7	0.26
RNA concentration (ng/mL)	53.6	44.0	0.16
RIN	8.2	8.6	0.90

EA = European-American, **AA** =African-American, **F** = Female, **M** = Male.
